# Surveillance for Cancers Associated with Tobacco Use — United States, 2010–2014

**DOI:** 10.15585/mmwr.ss6712a1

**Published:** 2018-11-02

**Authors:** M. Shayne Gallaway, S. Jane Henley, C. Brooke Steele, Behnoosh Momin, Cheryll C. Thomas, Ahmed Jamal, Katrina F. Trivers, Simple D. Singh, Sherri L. Stewart

**Affiliations:** 1 Division of Cancer Prevention and Control, National Center for Chronic Disease Prevention and Health Promotion, CDC; 2 Commissioned Corps, U.S. Public Health Service, Rockville, Maryland; 3 Office on Smoking and Health, National Center for Chronic Disease and Prevention and Health Promotion, CDC

## Abstract

**Problem/Condition:**

Tobacco use is the leading preventable cause of cancer, contributing to at least 12 types of cancer, including acute myeloid leukemia (AML) and cancers of the oral cavity and pharynx; esophagus; stomach; colon and rectum; liver; pancreas; larynx; lung, bronchus, and trachea; kidney and renal pelvis; urinary bladder; and cervix. This report provides a comprehensive assessment of recent tobacco-associated cancer incidence for each cancer type by sex, age, race/ethnicity, metropolitan county classification, tumor characteristics, U.S. census region, and state. These data are important for initiation, monitoring, and evaluation of tobacco prevention and control measures.

**Period Covered:**

2010–2014.

**Description of System:**

Cancer incidence data from CDC’s National Program of Cancer Registries and the National Cancer Institute’s Surveillance, Epidemiology, and End Results program were used to calculate average annual age-adjusted incidence rates for 2010–2014 and trends in annual age-adjusted incidence rates for 2010–2014. These cancer incidence data cover approximately 99% of the U.S. population. This report provides age-adjusted cancer incidence rates for each of the 12 cancer types known to be causally associated with tobacco use, including liver and colorectal cancer, which were deemed to be causally associated with tobacco use by the U.S. Surgeon General in 2014. Findings are reported by demographic and geographic characteristics, percentage distributions for tumor characteristics, and trends in cancer incidence by sex.

**Results:**

During 2010–2014, approximately 3.3 million new tobacco-associated cancer cases were reported in the United States, approximately 667,000 per year. Age-adjusted incidence rates ranged from 4.2 AML cases per 100,000 persons to 61.3 lung cancer cases per 100,000 persons. By cancer type, incidence rates were higher among men than women (excluding cervical cancer), higher among non-Hispanics than Hispanics (for all cancers except stomach, liver, kidney, and cervical), higher among persons in nonmetropolitan counties than those in metropolitan counties (for all cancers except stomach, liver, pancreatic, and AML), and lower in the West than in other U.S. census regions (all except stomach, liver, bladder, and AML). Compared with other racial/ethnic groups, certain cancer rates were highest among whites (oral cavity and pharyngeal, esophageal, bladder, and AML), blacks (colon and rectal, pancreatic, laryngeal, lung and bronchial, cervical, and kidney), and Asians/Pacific Islanders (stomach and liver). During 2010–2014, the rate of all tobacco-associated cancers combined decreased 1.2% per year, influenced largely by decreases in cancers of the larynx (3.0%), lung (2.2%), colon and rectum (2.1%), and bladder (1.3%).

**Interpretation:**

Although tobacco-associated cancer incidence decreased overall during 2010–2014, the incidence remains high in several states and subgroups, including among men, whites, blacks, non-Hispanics, and persons in nonmetropolitan counties. These disproportionately high rates of tobacco-related cancer incidence reflect overall demographic patterns of cancer incidence in the United States and also reflect patterns of tobacco use.

**Public Health Action:**

Tobacco-associated cancer incidence can be reduced through prevention and control of tobacco use and comprehensive cancer-control efforts focused on reducing cancer risk, detecting cancer early, and better assisting communities disproportionately affected by cancer. Ongoing surveillance to monitor cancer incidence can identify populations with a high incidence of tobacco-associated cancers and evaluate the effectiveness of tobacco control programs and policies. Implementation research can be conducted to achieve wider adoption of existing evidence-based cancer prevention and screening programs and tobacco control measures, especially to reach groups with the largest disparities in cancer rates.

## Introduction

Tobacco use is the leading cause of preventable disease and death in the United States, and approximately 480,000 deaths per year are caused by cigarette smoking and secondhand smoke exposure, or nearly one in five deaths annually (*1*,*2*). This includes approximately 41,000 deaths among adults and 400 deaths among infants resulting from secondhand smoke exposure (*1*,*3*,*4*). Tobacco smoke contains approximately 7,000 chemicals, including hundreds that are toxic. Approximately 70 of these chemicals can cause cancer (*1*,*5*). Forms of tobacco used in the United States include cigarettes, cigars, smokeless tobacco (i.e., chewing tobacco, snuff, dip, snus, and dissolvable tobacco), pipes, hookah (water pipes), bidis, and electronic cigarettes. In addition, smoking accounts for approximately $300 billion annually in direct health care expenditures ($170 billion) and lost productivity ($156 billion) (*1*,*6*).

The relation between smoking and lung cancer was first classified as causal in a landmark report released by the U.S. Surgeon General in 1964 (*7*). Subsequent Surgeon General reports have concluded that smoking causes acute myeloid leukemia (AML) and cancer in many other organ sites, including the cervix, esophagus, kidney and renal pelvis, larynx, trachea, lung and bronchus, oral cavity and pharynx (OCP), pancreas, stomach, and urinary bladder (*2*,*7*–*18*). Surgeon General reports also have concluded that the use of smokeless tobacco (i.e., snuff and chewing tobacco) causes cancers of the OCP and esophagus; cigar use causes cancers of the oral cavity, esophagus, larynx, and lung; and secondhand smoke exposure causes lung cancer (*19*). During 1999–2004, approximately 2.4 million new cases of tobacco-associated cancer were reported in the United States (*19*). In 2014, the Surgeon General expanded the list of cancer sites to include the liver, colon, and rectum (*1*). Approximately 30% of cancer deaths in the United States, including approximately 80% of lung cancer deaths, are attributable to tobacco (*1*,*2*,*20*–*23*). The International Agency for Research on Cancer has drawn conclusions (*24*–*28*) similar to the findings in the Surgeon General’s reports on tobacco and health.

When the first Surgeon General’s report on the health hazards of smoking was released in 1964, the prevalence of cigarette smoking in the United States was 42% but has since decreased (*1*). During 2005–2016, the prevalence of cigarette smoking among U.S. adults decreased from 20.9% to 15.5%, and the proportion of ever smokers (i.e., persons who have smoked at least 100 cigarettes during their lifetime) who quit increased from 50.8% to 59.0% (*29*). Despite this progress, the United States has not yet achieved the *Healthy People 2020* target of reducing the proportion of U.S. adults aged ≥18 years who smoke cigarettes to ≤12.0% (*1*,*30*). Moreover, large disparities in tobacco use remain across populations defined by race/ethnicity, sociodemographic status, U.S. census region, disability or limitation status, sexual orientation, and presence of serious psychological distress (*1*,*29*,*31*).

Furthermore, increases have occurred in the use of other tobacco products and the use of multiple products among youths. In 2016, among current tobacco product users, 47.2% of high school students and 42.4% of middle school students used two or more tobacco products, and electronic cigarettes were the most commonly used tobacco products among high school (11.3%) and middle school (4.3%) students (*32*). Since 2000, the prevalence of smokeless tobacco use in the United States has increased among adult males (6.7%) (*1*,*33*) and males in high school (8.3%) (*1*,*32*).

At least half of persons who smoke cigarettes for 20 years are expected to die from a tobacco-related disease, although tobacco cessation significantly decreases this risk (*34*–*36*). Therefore, among the 37.8 million persons in the United States who were current cigarette smokers in 2016 (*29*), approximately 18.9 million persons might die prematurely from a tobacco-related disease, including 6 million from cancer. Many tobacco-associated cancers could be prevented by population-level reduction of tobacco use through sustained, comprehensive state tobacco control programs. Proven population-based interventions, including increased tobacco prices, comprehensive smoke-free laws, antitobacco mass media campaigns, and barrier-free access to cessation assistance are critical to reduce cigarette smoking and smoking-related disease and deaths among U.S. adults (*29*). To help evaluate the effectiveness of tobacco control programs and policies and identify populations at greatest risk for developing cancers associated with tobacco use, ongoing surveillance of these cancers is essential.

This report describes the incidence of tobacco-associated cancers in the United States by cancer type, demographic and tumor characteristics, and state and U.S. census region. In addition, recent trends in cancer incidence are described. Cancer incidence data enable public health professionals to more effectively identify needs for cancer prevention and control at the national, state, and local levels (*37*–*39*). These data can be used to develop public health actions to reduce disparities in cancer outcomes (*40*) and to help measure the effectiveness of state-level tobacco control strategies such as tobacco-price increase implementation and smoke-free laws (*41*).

## Methods

To describe the incidence of tobacco-associated cancers in the United States, cancer incidence data from CDC’s National Program of Cancer Registries (NPCR) and the National Cancer Institute’s (NCI’s) Surveillance, Epidemiology, and End Results (SEER) program (*39*) were analyzed. Each year, NPCR- and SEER-funded central registries submit data on cancer diagnosed during the most recent year to the respective program. Rigorous quality-control edits, data completeness evaluations, and data quality assessment are performed on all data, and a registry’s data must meet multiple criteria (i.e., case ascertainment, missing information, and required fields) to be included in *U.S. Cancer Statistics*. The average annual age-adjusted rates and trends in rates for the most recent 5-year period with available data (2010–2014) are presented and cover 100% of the U.S. population. Combined data from the NPCR and SEER programs provide the best source of information on population-based cancer incidence for the United States (*42*).

### Incidence Data

Data on new cases of cancer diagnosed during 2010–2014 were obtained from 51 population-based cancer registries affiliated with NPCR and SEER programs in each state, the District of Columbia (DC), and Puerto Rico. Data from all registries except Nevada met *U.S. Cancer Statistics* publication criteria (*43*) for all years during 2010–2014. Data for Nevada were excluded from all analyses. Data for Puerto Rico are included in state-specific analyses but not in U.S. census region analyses. Cases were first classified by anatomic site according to the *International Classification of Diseases for Oncology, Third Edition* (ICD-O-3) (*44*), and cases with hematopoietic histologies were classified further using the 2008 *WHO Classification of Tumours of Haematopoietic and Lymphoid Tissues* (*45*). Only cases of invasive cancer were included for all analyses, except for urinary bladder cancer, for which in situ tumors also were included.

Tobacco-associated cancers were defined as those classified by the U.S. Surgeon General as causally related to cigarette smoking (*1*). These include AML (ICD-O-3 histology codes: 9840, 9861, 9865–9867, 9869, 9871–9874, 9895–9898, 9910–9911, and 9920) and cancers of the OCP (C00.0–C14.8); esophagus (C15.0–C15.9); stomach (C16.0–16.9); colon and rectum (C18.0–20.9 and C26.0); liver (C22.0); pancreas (C25.0–25.9); larynx (C32.0–32.9); trachea, lung, and bronchus (C33.9–34.9); cervix (C53.0–53.9); kidney and renal pelvis (C64.9–65.9); and urinary bladder (C67.0–67.9). Anatomic sites were restricted to cancers with histology codes 8000–9049, 9056–9139, and 9141–9589 (excluding mesothelioma, Kaposi sarcoma, and hematopoietic cancers). Because information on tobacco use was not routinely collected by all cancer registries, cases of all cancer classified as causal by the U.S. Surgeon General (*1*) are reported. Therefore, cases of cancer included in this report might or might not be in persons who used tobacco.

### Demographic and Tumor Characteristics

Incidence rates were estimated by several demographic characteristics including sex, age, race/ethnicity, and U.S. census region. Age was categorized as <40, 40–49, 50–59, 60–69, 70–79, and ≥80 years. Information about race/ethnicity was collected from medical records; race was classified as white, black, American Indian/Alaska Native (AI/AN), and Asian/Pacific Islander (A/PI) and ethnicity as Hispanic or non-Hispanic. Cases among persons with other or unknown race (2%) or unknown ethnicity (2%) were included in overall rates but were not included as separate categories because population denominators were not available. U.S. census regions included the Northeast (Connecticut, Maine, Massachusetts, New Hampshire, New Jersey, New York, Pennsylvania, Rhode Island, and Vermont); Midwest (Illinois, Indiana, Iowa, Kansas, Michigan, Minnesota, Missouri, Nebraska, North Dakota, Ohio, South Dakota, and Wisconsin); South (Alabama, Arkansas, DC, Delaware, Florida, Georgia, Kentucky, Louisiana, Maryland, Mississippi, North Carolina, Oklahoma, South Carolina, Tennessee, Texas, Virginia, and West Virginia); and West (Alaska, Arizona, California, Colorado, Hawaii, Idaho, Montana, New Mexico, Oregon, Utah, Washington, and Wyoming).

For some cancer sites, several tumor characteristics are described, including histology, anatomic subsite (i.e., a specific location within a cancer site, such as the lip [subsite] for OCP [site]), and stage at diagnosis. Histologic groups for each cancer site were determined by incidence or clinical relevance. SEER Summary Stage 2000 was used to characterize stage at diagnosis as localized (cancer that is confined to the primary site), regional (cancer that has spread directly beyond the primary site or to regional lymph nodes), distant (cancer that has spread to other organs), or unknown stage using clinical and pathologic tumor characteristics, such as tumor size, depth of invasion and extension to regional or distant tissues, involvement of regional lymph nodes, and distant metastases (https://seer.cancer.gov/tools/ssm). Analyses by tumor characteristics excluded cases that were reported only by death certificate or autopsy and cases that were not microscopically confirmed (except for liver cancer because a large percentage of these cancers [29%] were confirmed by radiography).

### Statistical Analysis

Population estimates for incidence rate denominators were a modification of annual county population estimates by age, sex, bridged race, and ethnicity produced by the U.S. Census Bureau in collaboration with CDC and with support from the National Cancer Institute (https://seer.cancer.gov/popdata). Incidence rates per 100,000 population were age adjusted to the 2000 U.S. standard population; 95% confidence intervals were calculated as modified gamma intervals (*46*) and are presented to allow for informal comparison among rates, without defining a referent group. Although using the overlap between confidence intervals to determine significance is conservative, the confidence intervals provide a measure of the variability in the rates and perspective for making comparisons. Trends in age-adjusted incidence rates of invasive cancer were examined (by site and sex [men, women, and combined]), and the annual percentage change (APC) was used to quantify changes in rates during 2010–2014 by using least-squares regression. A t-test was used to determine whether an APC was significantly different from zero. The APC and associated p value corresponds to the overall increasing or decreasing trends observed during this time. Rates were considered to increase if the APC >0 (p<0.05) and to decrease if the APC <0 (p<0.05); otherwise, rates were considered stable. State-specific age-adjusted tobacco-associated cancer incidence rates were mapped using quartiles as cut points.

## Results

### Lung Cancer

**FIGURE 1:**
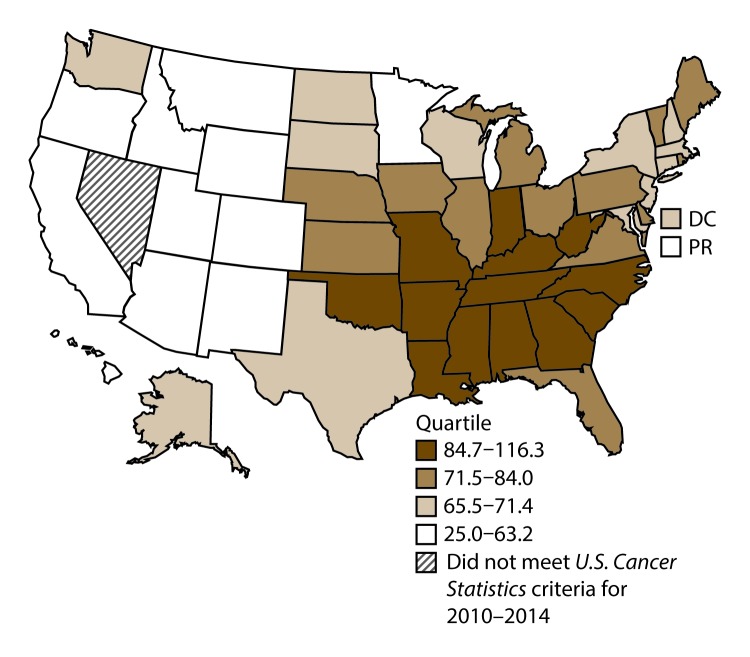
Incidence rates* for male lung, bronchial, and tracheal cancers, by state/area and U.S. census region^†^ — United States,^§^ 2010–2014 **Abbreviations:** DC = District of Columbia; PR = Puerto Rico. * New cases diagnosed per 100,000 males, age adjusted to the 2000 U.S. standard population. ^†^ *West:* 53.5; *Midwest:* 78.6; *Northeast:* 72.0; *South:* 80.8. (*West:* Alaska, Arizona, California, Colorado, Hawaii, Idaho, Montana, Oregon, New Mexico, Utah, Washington, and Wyoming; *Midwest:* Illinois, Indiana, Iowa, Kansas, Michigan, Minnesota, Missouri, Nebraska, North Dakota, Ohio, South Dakota, and Wisconsin; *Northeast:* Connecticut, Maine, Massachusetts, New Hampshire, New Jersey, New York, Pennsylvania, Rhode Island, and Vermont; *South:* Alabama, Arkansas, Delaware, District of Columbia, Florida, Georgia, Kentucky, Louisiana, Maryland, Mississippi, North Carolina, Oklahoma, South Carolina, Tennessee, Texas, Virginia, and West Virginia.) ^§^ Cancer incidence data were compiled from cancer registries that met the data quality criteria for all invasive cancer sites combined, representing approximately 99% of the U.S. population. (Data from Nevada did not meet *U.S. Cancer Statistics* publication criteria for 2010–2014.) Data for Puerto Rico are included in state-specific analyses but not in U.S. census region analyses.

**FIGURE 2:**
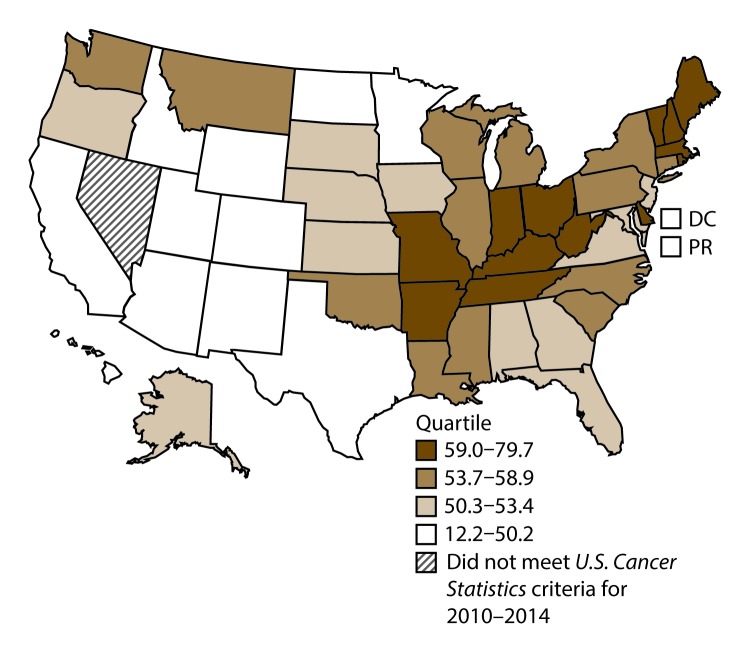
Incidence rates* for female lung, bronchial, and tracheal cancers, by state/area and U.S. census region^†^ — United States,^§^ 2010–2014 **Abbreviations:** DC = District of Columbia; PR = Puerto Rico. * New cases diagnosed per 100,000 females, age adjusted to the 2000 U.S. standard population. ^†^ *West:* 42.7; *Midwest:* 57.4; *Northeast:* 56.0; *South:* 53.8. (*West:* Alaska, Arizona, California, Colorado, Hawaii, Idaho, Montana, Oregon, New Mexico, Utah, Washington, and Wyoming; *Midwest:* Illinois, Indiana, Iowa, Kansas, Michigan, Minnesota, Missouri, Nebraska, North Dakota, Ohio, South Dakota, and Wisconsin; *Northeast:* Connecticut, Maine, Massachusetts, New Hampshire, New Jersey, New York, Pennsylvania, Rhode Island, and Vermont; *South:* Alabama, Arkansas, Delaware, District of Columbia, Florida, Georgia, Kentucky, Louisiana, Maryland, Mississippi, North Carolina, Oklahoma, South Carolina, Tennessee, Texas, Virginia, and West Virginia.) ^§^ Cancer incidence data were compiled from cancer registries that met the data quality criteria for all invasive cancer sites combined, representing approximately 99% of the U.S. population. (Data from Nevada did not meet *U.S. Cancer Statistics* publication criteria for 2010–2014.) Data for Puerto Rico are included in state-specific analyses but not in U.S. census region analyses.

A total of 1,070,504 new cases (61.3 per 100,000 persons) of cancers of the lung, bronchus, and trachea (lung cancer) were reported in the United States during 2010–2014 (Table 1). Incidence rates were substantially higher among men (72.7) than among women (52.7). Rates increased with increasing age and peaked among persons aged 70–79 years (390.0). Among men, blacks had the highest rates (85.9), followed by whites (72.4), AI/ANs (52.2), and A/PIs (45.1). Among women, whites had the highest rates (54.3), followed by blacks (49.2), AI/ANs (39.0), and A/PIs (27.9). Rates were two times higher among non-Hispanics than among Hispanics (64.1 versus 31.9, respectively). Among those with known tumor characteristics (88.1%), approximately 81% of all lung cancer cases were non–small cell carcinomas. Adenocarcinoma was the most common histologic subtype, although women had greater percentages of adenocarcinomas than men (52.4% and 43.2%, respectively). Men had greater percentages of squamous cell carcinoma than women (28.9% and 19.4%, respectively). More than half (52.6%) of lung cancer cases were diagnosed at the distant stage. During 2010–2014, lung cancer incidence rates were higher in nonmetropolitan counties (69.4) than in metropolitan counties (59.8). Among men, rates were highest in the South (80.8) and lowest in the West (53.5). Among women, rates were highest in the Midwest (57.4) and lowest in the West (42.7). Kentucky and West Virginia had some of the highest rates both among men (98.8–116.3) and women (66.3–79.7) (Figures 1 and 2).

**TABLE 1:** Incidence rates* and percentages^†^ of invasive lung, bronchial, and tracheal cancers, by demographic and tumor characteristics — United States,^§^ 2010–2014

Demographic characteristic	Total		Male		Female	
	No.	Rate (95% CI)	No.	Rate (95% CI)	No.	Rate (95% CI)
**Total**	**1,070,504**	**61.3 (61.2–61.4)**	**566,713**	**72.7 (72.5–72.9)**	**503,791**	**52.7 (52.5–52.8)**
**Age group at diagnosis (yrs)**						
<40	5,413	0.7 (0.7–0.7)	2,624	0.7 (0.7–0.7)	2,789	0.7 (0.7–0.8)
40–49	34,419	15.6 (15.5–15.8)	16,262	14.9 (14.7–15.2)	18,157	16.4 (16.1–16.6)
50–59	163,296	74.3 (74.0–74.7)	87,194	81.3 (80.7–81.8)	76,102	67.8 (67.3–68.3)
60–69	313,543	202.8 (202.1–203.5)	172,045	234.2 (233.1–235.3)	141,498	174.4 (173.5–175.3)
70–79	340,129	390.0 (388.7–391.3)	180,917	461.9 (459.8–464.0)	159,212	331.6 (330.0–333.3)
≥80	213,704	374.5 (372.9–376.1)	107,671	502.7 (499.7–505.7)	106,033	298.6 (296.7–300.4)
**Race**						
White	920,427	62.1 (61.9–62.2)	483,499	72.4 (72.2–72.6)	436,928	54.3 (54.1–54.4)
Black	113,633	64.0 (63.6–64.4)	63,233	85.9 (85.2–86.7)	50,400	49.2 (48.7–49.6)
American Indian/Alaska Native	6,000	44.7 (43.5–45.9)	3,111	52.2 (50.2–54.2)	2,889	39.0 (37.6–40.6)
Asian/Pacific Islander	25,905	35.2 (34.8–35.6)	14,286	45.1 (44.3–45.9)	11,619	27.9 (27.4–28.4)
**Ethnicity^¶^**						
Hispanic	44,500	31.9 (31.6–32.2)	24,297	40.8 (40.2–41.3)	20,203	25.5 (25.2–25.9)
Non-Hispanic	1,025,933	64.1 (64.0–64.2)	542,388	75.7 (75.5–75.9)	483,545	55.3 (55.1–55.4)
**County classification**						
Metropolitan	841,631	59.8 (59.7–59.9)	438,447	70.4 (70.1–70.6)	403,184	51.9 (51.8–52.1)
Nonmetropolitan	178,536	69.4 (69.0–69.7)	100,611	84.9 (84.4–85.4)	77,925	57.0 (56.6–57.4)
**Census region**						
Northeast	208,137	62.6 (62.3–62.9)	104,465	72.0 (71.5–72.4)	103,672	56.0 (55.7–56.4)
Midwest	258,247	66.5 (66.2–66.7)	136,591	78.6 (78.2–79.0)	121,656	57.4 (57.1–57.7)
South	430,881	65.5 (65.3–65.7)	237,375	80.8 (80.4–81.1)	193,506	53.8 (53.6–54.0)
West	173,239	47.4 (47.2–47.6)	88,282	53.5 (53.1–53.8)	84,957	42.7 (42.4–43.0)
Tumor characteristic**	**No.**	**%**	**No.**	**%**	**No.**	**%**
**Total**	**942,919**	**100.0**	**498,902**	**100.0**	**444,017**	**100.0**
**Histology**						
Non–small cell carcinoma	764,914	81.1	408,889	81.9	356,025	80.1
Adenocarcinoma	448,320	47.5	215,628	43.2	232,692	52.4
Squamous cell carcinoma	230,569	24.5	144,310	28.9	86,259	19.4
Non–small cell carcinoma, NOS	70,142	7.4	39,900	8.0	30,242	6.8
Large cell carcinoma	15,883	1.7	9,051	1.8	6,832	1.5
Small cell carcinoma	133,192	14.1	65,925	13.2	67,267	15.1
Epithelial carcinoma	23,319	2.5	13,540	2.7	9,779	2.2
All other histologies	21,494	2.3	10,548	2.1	10,946	2.5
**Stage^††^**						
Localized	189,113	20.1	89,436	17.9	99,677	22.4
Regional	227,876	24.2	120,567	24.2	107,309	24.2
Distant	495,671	52.6	272,506	54.6	223,165	50.3
Unknown	30,259	3.2	16,393	3.3	13,866	3.1

**Abbreviations:** CI = confidence interval; NOS = not otherwise specified.

* New cases diagnosed per 100,000 persons, age adjusted to the 2000 U.S. standard population.

^†^ New cases diagnosed.

^§^ Cancer incidence data were compiled from cancer registries that met the data quality criteria for all invasive cancer sites combined, representing approximately 99% of the U.S. population. (Data from Nevada did not meet *U.S. Cancer Statistics* publication criteria for 2010–2014.)

^¶^ Ethnicity is not mutually exclusive from race.

** Only includes microscopically confirmed cases; excludes cases identified only through death certificate or autopsy report.

^††^ Localized: cancer that is confined to the primary site; regional: cancer that has spread directly beyond the primary site or to regional lymph nodes; distant: cancer that has spread to other organs.

### Laryngeal Cancer

**FIGURE 3:**
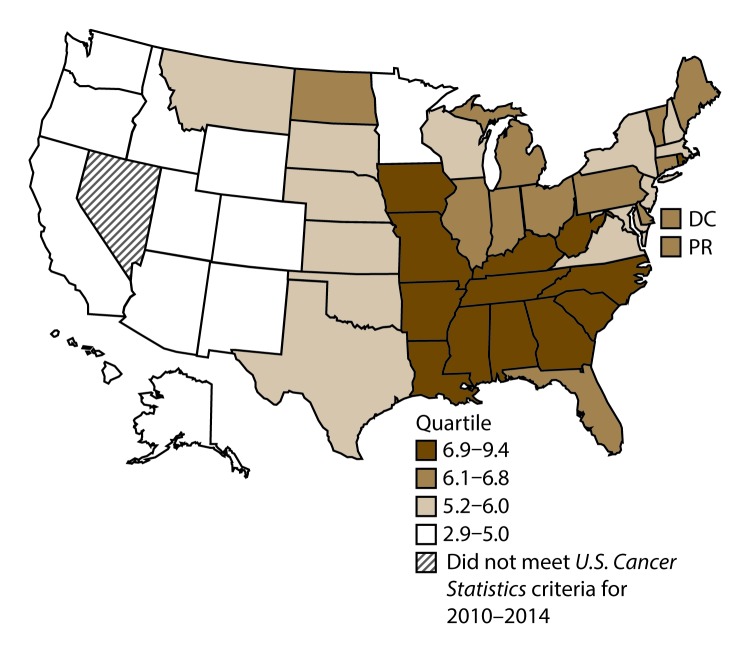
Incidence rates* for male laryngeal cancer, by state/area and U.S. census region^†^ — United States,^§^ 2010–2014 **Abbreviations:** DC = District of Columbia; PR = Puerto Rico. * New cases diagnosed per 100,000 males, age adjusted to the 2000 U.S. standard population. ^†^ *West:* 4.0; *Midwest:* 6.4; *Northeast:* 6.1; *South:* 6.8. (*West:* Alaska, Arizona, California, Colorado, Hawaii, Idaho, Montana, Oregon, New Mexico, Utah, Washington, and Wyoming; *Midwest:* Illinois, Indiana, Iowa, Kansas, Michigan, Minnesota, Missouri, Nebraska, North Dakota, Ohio, South Dakota, and Wisconsin; *Northeast:* Connecticut, Maine, Massachusetts, New Hampshire, New Jersey, New York, Pennsylvania, Rhode Island, and Vermont; *South:* Alabama, Arkansas, Delaware, District of Columbia, Florida, Georgia, Kentucky, Louisiana, Maryland, Mississippi, North Carolina, Oklahoma, South Carolina, Tennessee, Texas, Virginia, and West Virginia.) ^§^ Cancer incidence data were compiled from cancer registries that met the data quality criteria for all invasive cancer sites combined, representing approximately 99% of the U.S. population. (Data from Nevada did not meet *U.S. Cancer Statistics* publication criteria for 2010–2014.) Data for Puerto Rico are included in state-specific analyses but not in U.S. census region analyses.

**FIGURE 4:**
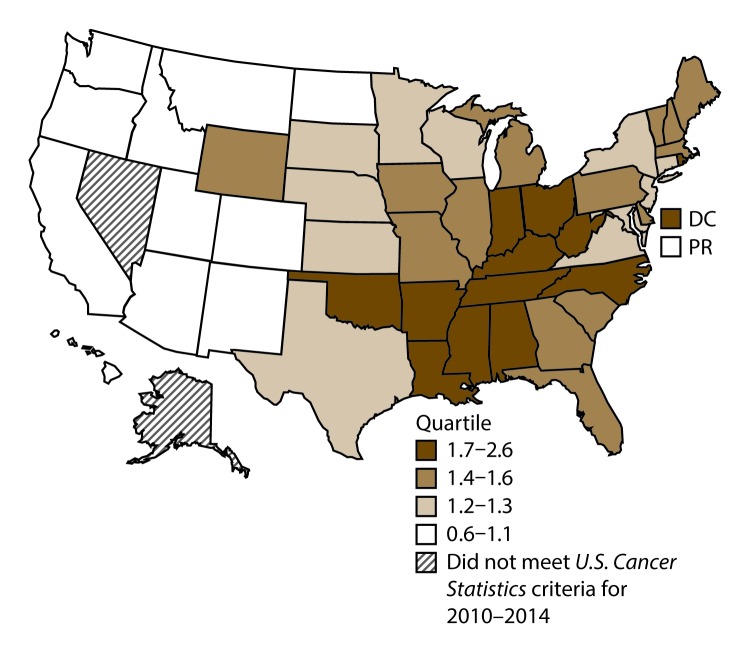
Incidence rates* for female laryngeal cancer, by state/ area and U.S. census region^†^ — United States,^§^ 2010–2014 **Abbreviations:** DC = District of Columbia; PR = Puerto Rico. * New cases diagnosed per 100,000 females, age adjusted to the 2000 U.S. standard population. ^†^ *West:* 0.8; *Midwest:* 1.6; *Northeast:* 1.4; *South:* 1.5. (*West:* Alaska, Arizona, California, Colorado, Hawaii, Idaho, Montana, Oregon, New Mexico, Utah, Washington, and Wyoming; *Midwest:* Illinois, Indiana, Iowa, Kansas, Michigan, Minnesota, Missouri, Nebraska, North Dakota, Ohio, South Dakota, and Wisconsin; *Northeast:* Connecticut, Maine, Massachusetts, New Hampshire, New Jersey, New York, Pennsylvania, Rhode Island, and Vermont; *South:* Alabama, Arkansas, Delaware, District of Columbia, Florida, Georgia, Kentucky, Louisiana, Maryland, Mississippi, North Carolina, Oklahoma, South Carolina, Tennessee, Texas, Virginia, and West Virginia.) ^§^ Cancer incidence data were compiled from cancer registries that met the data quality criteria for all invasive cancer sites combined, representing approximately 99% of the U.S. population. (Data from Nevada did not meet *U.S. Cancer Statistics* publication criteria for 2010–2014.) Data for Puerto Rico are included in state-specific analyses but not in U.S. census region analyses.

A total of 62,479 new cases (3.5 per 100,000 persons) of laryngeal cancer were reported in the United States during 2010–2014 (Table 2). Incidence rates were substantially higher among men (6.0) than among women (1.3). Rates increased with increasing age and peaked among persons aged 70–79 years (16.2). Among men, blacks had the highest rates (8.5), followed by whites (5.9), AI/ANs (3.6), and A/PIs (2.2). Rates were higher among non-Hispanics than among Hispanics (3.6 and 2.5, respectively). Among those with known tumor characteristics (97.1%), almost all (96.9%) laryngeal cancers were squamous cell carcinomas. The majority (53.6%) were diagnosed at a localized stage; however, a smaller percentage of localized cases occurred among women (47.1%) than men (55.3%). Women had a greater percentage of regional stage laryngeal cancer diagnoses than men (32.2% and 22.0%, respectively), whereas men had slightly greater percentages of distant stage diagnoses than women (17.9% for men and 16.5% for women). During 2010–2014, laryngeal cancer incidence rates were higher in nonmetropolitan counties (4.2) than in metropolitan counties (3.3). Among men, rates were highest in the South region of the United States (6.8) and lowest in the West (4.0). Among women, rates were similar in the South, Midwest, and Northeast (1.4–1.6) and were lower in the West (0.8). Kentucky, Louisiana, Mississippi, and West Virginia had some of the highest rates both among men (8.3–9.4) and women (1.9–2.6) (Figures 3 and 4).

**TABLE 2:** Incidence rates* and percentages^†^ of invasive laryngeal cancer, by demographic and tumor characteristics — United States,^§^ 2010–2014

Demographic characteristic	Total		Male		Female	
	No.	Rate (95% CI)	No.	Rate (95% CI)	No.	Rate (95% CI)
**Total**	**62,479**	**3.5 (3.4–3.5)**	**49,605**	**6.0 (6.0–6.1)**	**12,874**	**1.3 (1.3–1.4)**
**Age group at diagnosis (yrs)**						
<40	563	0.1 (0.1–0.1)	374	0.1 (0.1–0.1)	189	0.0 (0.0–0.1)
40–49	3,888	1.8 (1.7–1.8)	2,855	2.6 (2.5–2.7)	1,033	0.9 (0.9–1.0)
50–59	16,028	7.3 (7.2–7.5)	12,437	11.7 (11.5–11.9)	3,591	3.2 (3.1–3.3)
60–69	20,840	13.4 (13.2–13.5)	16,802	22.7 (22.3–23.0)	4,038	4.9 (4.8–5.1)
70–79	14,176	16.2 (15.9–16.4)	11,470	29.1 (28.6–29.6)	2,706	5.6 (5.4–5.8)
≥80	6,984	12.2 (12.0–12.5)	5,667	26.5 (25.8–27.2)	1,317	3.7 (3.5–3.9)
**Race**						
White	51,997	3.4 (3.4–3.5)	41,239	5.9 (5.8–5.9)	10,758	1.4 (1.3–1.4)
Black	8,751	4.5 (4.4–4.6)	6,911	8.5 (8.3–8.7)	1,840	1.7 (1.6–1.7)
American Indian/Alaska Native	331	2.1 (1.8–2.3)	260	3.6 (3.1–4.1)	71	0.8 (0.6–1.0)
Asian/Pacific Islander	878	1.1 (1.1–1.2)	752	2.2 (2.1–2.4)	126	0.3 (0.2–0.3)
**Ethnicity^¶^**						
Hispanic	3,865	2.5 (2.4–2.6)	3,299	4.8 (4.7–5.0)	566	0.7 (0.6–0.7)
Non-Hispanic	58,606	3.6 (3.5–3.6)	46,300	6.1 (6.1–6.2)	12,306	1.4 (1.4–1.4)
**County classification**						
Metropolitan	48,624	3.3 (3.3–3.4)	38,562	5.8 (5.7–5.9)	10,062	1.3 (1.3–1.3)
Nonmetropolitan	10,920	4.2 (4.2–4.3)	8,685	7.1 (6.9–7.3)	2,235	1.7 (1.6–1.8)
**Census region**						
Northeast	11,835	3.5 (3.4–3.5)	9,320	6.1 (6.0–6.2)	2,515	1.4 (1.3–1.4)
Midwest	15,154	3.8 (3.7–3.9)	11,845	6.4 (6.3–6.5)	3,309	1.6 (1.5–1.6)
South	26,865	4.0 (3.9–4.0)	21,346	6.8 (6.8–6.9)	5,519	1.5 (1.5–1.6)
West	8,625	2.3 (2.2–2.3)	7,094	4.0 (3.9–4.1)	1,531	0.8 (0.7–0.8)
Tumor characteristic**	**No.**	**%**	**No.**	**%**	**No.**	**%**
**Total**	**60,676**	**100.0**	**48,165**	**100.0**	**12,511**	**100.0**
**Histology**						
Squamous cell carcinoma	58,810	96.9	46,767	97.1	12,043	96.3
Adenocarcinoma	381	0.6	238	0.5	143	1.1
Epithelial carcinoma, NOS	881	1.5	687	1.4	194	1.6
All other histologies	604	1.0	473	1.0	131	1.0
**Stage^††^**						
Localized	32,534	53.6	26,646	55.3	5,888	47.1
Regional	14,640	24.1	10,608	22.0	4,032	32.2
Distant	10,702	17.6	8,632	17.9	2,070	16.5
Unknown	2,800	4.6	2,279	4.7	521	4.2

**Abbreviations:** CI = confidence interval; NOS = not otherwise specified.

* New cases diagnosed per 100,000 persons, age adjusted to the 2000 U.S. standard population.

^†^ New cases diagnosed.

^§^ Cancer incidence data were compiled from cancer registries that met the data quality criteria for all invasive cancer sites combined, representing approximately 99% of the U.S. population. (Data from Nevada did not meet *U.S. Cancer Statistics* publication criteria for 2010–2014.)

^¶^ Ethnicity is not mutually exclusive from race.

** Only includes microscopically confirmed cases; excludes cases identified only through death certificate or autopsy report.

^††^ Localized: cancer that is confined to the primary site; regional: cancer that has spread directly beyond the primary site or to regional lymph nodes; distant: cancer that has spread to other organs.

### Oral Cavity and Pharyngeal Cancer

**FIGURE 5:**
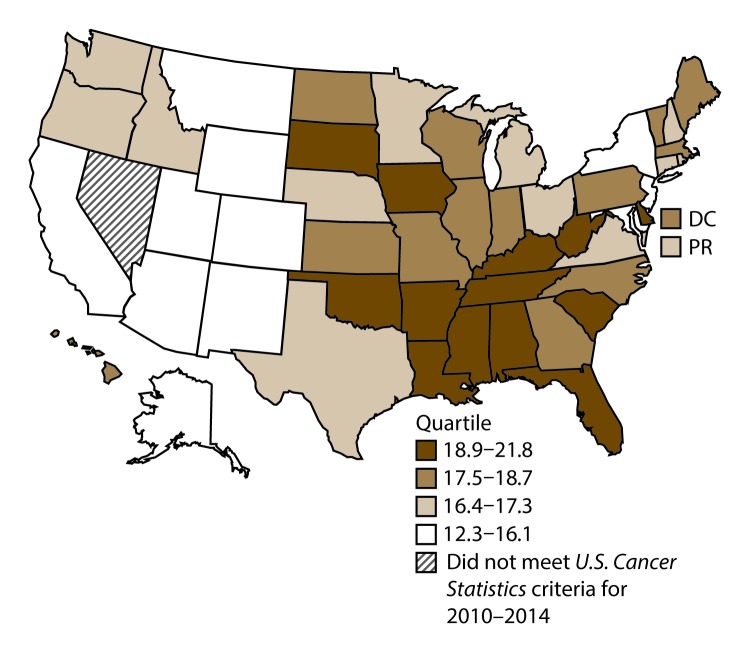
Incidence rates* for male oral cavity and pharyngeal cancers, by state/area and U.S. census region^†^ — United States,^§^ 2010–2014 **Abbreviations:** DC = District of Columbia; PR = Puerto Rico. * New cases diagnosed per 100,000 males, age adjusted to the 2000 U.S. standard population. ^†^ *West:* 15.5; *Midwest:* 17.7; *Northeast:* 16.6; *South:* 18.6. (*West:* Alaska, Arizona, California, Colorado, Hawaii, Idaho, Montana, Oregon, New Mexico, Utah, Washington, and Wyoming; *Midwest:* Illinois, Indiana, Iowa, Kansas, Michigan, Minnesota, Missouri, Nebraska, North Dakota, Ohio, South Dakota, and Wisconsin; *Northeast:* Connecticut, Maine, Massachusetts, New Hampshire, New Jersey, New York, Pennsylvania, Rhode Island, and Vermont; *South:* Alabama, Arkansas, Delaware, District of Columbia, Florida, Georgia, Kentucky, Louisiana, Maryland, Mississippi, North Carolina, Oklahoma, South Carolina, Tennessee, Texas, Virginia, and West Virginia.) ^§^ Cancer incidence data were compiled from cancer registries that met the data quality criteria for all invasive cancer sites combined, representing approximately 99% of the U.S. population. (Data from Nevada did not meet *U.S. Cancer Statistics* publication criteria for 2010–2014.) Data for Puerto Rico are included in state-specific analyses but not in U.S. census region analyses.

**FIGURE 6:**
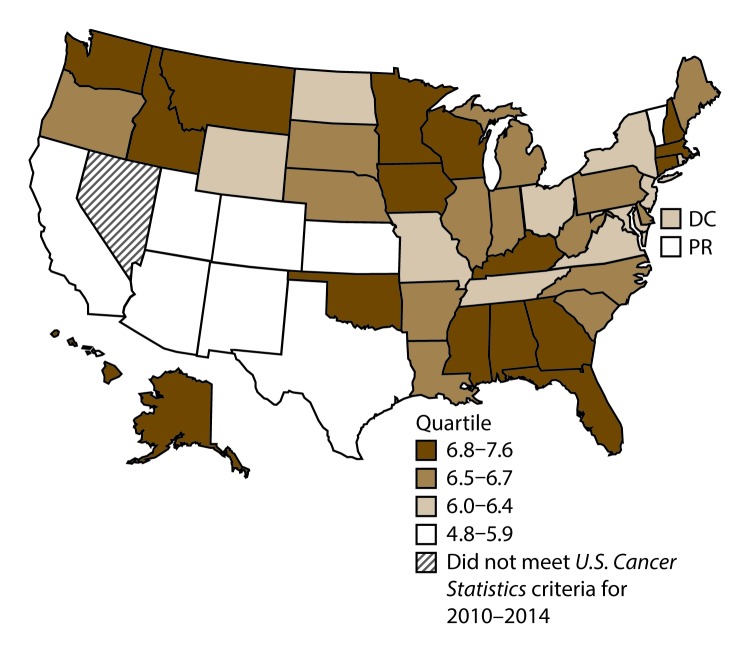
Incidence rates* for female oral cavity and pharyngeal cancers, by state/area and U.S. census region^†^ — United States,^§^ 2010–2014 **Abbreviations:** DC = District of Columbia; PR = Puerto Rico. * New cases diagnosed per 100,000 females, age adjusted to the 2000 U.S. standard population. ^†^ *West:* 5.9; *Midwest:* 6.5; *Northeast:* 6.5; *South:* 6.5. (*West:* Alaska, Arizona, California, Colorado, Hawaii, Idaho, Montana, Oregon, New Mexico, Utah, Washington, and Wyoming; *Midwest:* Illinois, Indiana, Iowa, Kansas, Michigan, Minnesota, Missouri, Nebraska, North Dakota, Ohio, South Dakota, and Wisconsin; *Northeast:* Connecticut, Maine, Massachusetts, New Hampshire, New Jersey, New York, Pennsylvania, Rhode Island, and Vermont; *South:* Alabama, Arkansas, Delaware, District of Columbia, Florida, Georgia, Kentucky, Louisiana, Maryland, Mississippi, North Carolina, Oklahoma, South Carolina, Tennessee, Texas, Virginia, and West Virginia.) ^§^ Cancer incidence data were compiled from cancer registries that met the data quality criteria for all invasive cancer sites combined, representing approximately 99% of the U.S. population. (Data from Nevada did not meet *U.S. Cancer Statistics* publication criteria for 2010–2014.) Data for Puerto Rico are included in state-specific analyses but not in U.S. census region analyses.

A total of 204,537 new cases (11.5 per 100,000 persons) of OCP cancer were reported in the United States during 2010–2014 (Table 3). Incidence rates were substantially higher among men (17.4) than among women (6.4). Rates increased with age and peaked among men aged 70–79 years (63.6) and women aged ≥80 years (29.4). Among men, whites had the highest rates (17.8), followed by blacks (14.5), A/PIs (11.2), and AI/ANs (10.9). Among women, whites had the highest rates (6.5), followed by blacks (5.1), A/PIs (5.0), and AI/ANs (4.1). Rates were higher among non-Hispanics than Hispanics (12.0 and 7.1, respectively). The most common subsites were the oral cavity (7.9) and tonsils (3.6) among men and the oral cavity (3.5) and salivary glands (1.0) among women. Among those with known tumor characteristics (97.5%), the majority of OCP cancers were squamous cell carcinomas (85.9%). Most were diagnosed at the localized (31.7%) and regional (45.3%) stages. However, a greater percentage of cases were diagnosed at the localized stage among women than men (43.0% and 27.1%, respectively), and a greater percentage of cases were diagnosed at the regional stage among men than women (49.0% and 36.3%, respectively). During 2010–2014, OCP cancer incidence rates were higher in nonmetropolitan counties (12.5) than in metropolitan counties (11.3). Among men, rates were highest in the South region of the United States (18.6) and lowest in the West (15.5). Among women, rates of OCP cancers were similar in the South, Midwest, and Northeast (6.5) and were lower in the West (5.9). Alabama, Arkansas, Florida, Kentucky, Louisiana, Mississippi, and Oklahoma had some of the highest rates both among men (19.9–21.8) and women (6.6–7.4) (Figures 5 and 6).

**TABLE 3:** Incidence rates* and percentages^†^ of invasive oral cavity and pharyngeal cancers, by demographic and tumor characteristics — United States,^§^ 2010–2014

Demographic characteristic	Total		Male		Female	
	No.	Rate (95% CI)	No.	Rate (95% CI)	No.	Rate (95% CI)
**Total**	**204,537**	**11.5 (11.4–11.5)**	**144,851**	**17.4 (17.3–17.4)**	**59,686**	**6.4 (6.3–6.4)**
**Age group at diagnosis (yrs)**						
<40	7,562	1.0 (0.9–1.0)	4,153	1.1 (1.0–1.1)	3,409	0.9 (0.8–0.9)
40–49	19,866	9.1 (9.0–9.3)	14,391	13.3 (13.1–13.6)	5,475	5.0 (4.9–5.1)
50–59	56,098	25.8 (25.6–26.0)	42,834	40.3 (40.0–40.7)	13,264	11.9 (11.7–12.1)
60–69	60,210	38.4 (38.1–38.7)	45,247	60.7 (60.1–61.2)	14,963	18.3 (18.0–18.6)
70–79	37,022	42.3 (41.8–42.7)	25,140	63.6 (62.8–64.4)	11,882	24.7 (24.3–25.2)
≥80	23,779	41.3 (40.8–41.8)	13,086	61.2 (60.1–62.3)	10,693	29.4 (28.8–29.9)
**Race**						
White	176,221	11.8 (11.8–11.9)	125,496	17.8 (17.7–17.9)	50,725	6.5 (6.4–6.5)
Black	18,089	9.2 (9.0–9.3)	12,574	14.5 (14.3–14.8)	5,515	5.1 (5.0–5.2)
American Indian/Alaska Native	1,191	7.3 (6.8–7.7)	843	10.9 (10.1–11.7)	348	4.1 (3.7–4.6)
Asian/Pacific Islander	6,519	7.8 (7.6–8.0)	4,257	11.2 (10.9–11.6)	2,262	5.0 (4.8–5.2)
**Ethnicity^¶^**						
Hispanic	11,854	7.1 (6.9–7.2)	8,096	10.5 (10.3–10.8)	3,758	4.2 (4.1–4.3)
Non-Hispanic	192,676	12.0 (12.0–12.1)	136,750	18.2 (18.1–18.3)	55,926	6.6 (6.6–6.7)
**County classification**						
Metropolitan	163,768	11.3 (11.2–11.3)	115,587	17.1 (17.0–17.2)	48,181	6.3 (6.2–6.3)
Nonmetropolitan	31,220	12.5 (12.3–12.6)	22,450	18.7 (18.4–18.9)	8,770	6.7 (6.6–6.9)
**Census region**						
Northeast	37,358	11.1 (11.0–11.3)	25,665	16.6 (16.4–16.8)	11,693	6.5 (6.4–6.6)
Midwest	46,294	11.7 (11.6–11.9)	32,792	17.7 (17.5–17.9)	13,502	6.5 (6.4–6.6)
South	80,910	12.1 (12.0–12.2)	58,190	18.6 (18.5–18.8)	22,720	6.5 (6.4–6.6)
West	39,975	10.4 (10.3–10.6)	28,204	15.5 (15.4–15.7)	11,771	5.9 (5.8–6.0)
**Anatomic subsite**						
Lip	10,035	0.6 (0.6–0.6)	7,284	0.9 (0.9–1.0)	2,751	0.3 (0.3–0.3)
Oral cavity	99,539	5.6 (5.5–5.6)	66,678	7.9 (7.9–8.0)	32,861	3.5 (3.4–3.5)
Salivary gland	21,517	1.3 (1.3–1.3)	12,629	1.7 (1.6–1.7)	8,888	1.0 (1.0–1.0)
Tonsil	38,061	2.1 (2.1–2.1)	31,175	3.6 (3.5–3.6)	6,886	0.7 (0.7–0.7)
Oropharynx	9,428	0.5 (0.5–0.5)	7,189	0.8 (0.8–0.9)	2,239	0.2 (0.2–0.2)
Nasopharynx	14,393	0.8 (0.8–0.8)	10,925	1.3 (1.3–1.3)	3,468	0.4 (0.4–0.4)
Hypopharynx	6,004	0.3 (0.3–0.3)	4,700	0.6 (0.5–0.6)	1,304	0.1 (0.1–0.1)
Other oral cavity and pharynx	5,560	0.3 (0.3–0.3)	4,271	0.5 (0.5–0.5)	1,289	0.1 (0.1–0.1)
Tumor characteristic**	**No.**	**%**	**No.**	**%**	**No.**	**%**
**Total**	**199,496**	**100.0**	**141,383**	**100.0**	**58,113**	**100.0**
**Histology**						
Squamous cell carcinoma	171,299	85.9	126,036	89.1	45,263	77.9
Adenocarcinoma	7,579	3.8	4,014	2.8	3,565	6.1
Mucoepidermoid carcinoma	6,259	3.1	2,802	2.0	3,457	5.9
Epithelial carcinoma, NOS	5,711	2.9	4,094	2.9	1,617	2.8
All other histologies	8,648	4.3	4,437	3.1	4,211	7.2
**Stage^††^**						
Localized	63,275	31.7	38,268	27.1	25,007	43.0
Regional	90,347	45.3	69,275	49.0	21,072	36.3
Distant	35,439	17.8	26,741	18.9	8,698	15.0
Unknown	10,435	5.2	7,099	5.0	3,336	5.7

**Abbreviations:** CI = confidence interval; NOS = not otherwise specified.

* New cases diagnosed per 100,000 persons, age adjusted to the 2000 U.S. standard population.

^†^ New cases diagnosed.

^§^ Cancer incidence data were compiled from cancer registries that met the data quality criteria for all invasive cancer sites combined, representing approximately 99% of the U.S. population. (Data from Nevada did not meet *U.S. Cancer Statistics* publication criteria for 2010–2014.)

^¶^ Ethnicity is not mutually exclusive from race.

** Only includes microscopically confirmed cases; excludes cases identified only through death certificate or autopsy report.

^††^ Localized: cancer that is confined to the primary site; regional: cancer that has spread directly beyond the primary site or to regional lymph nodes; distant: cancer that has spread to other organs.

### Esophageal Cancer

**FIGURE 7:**
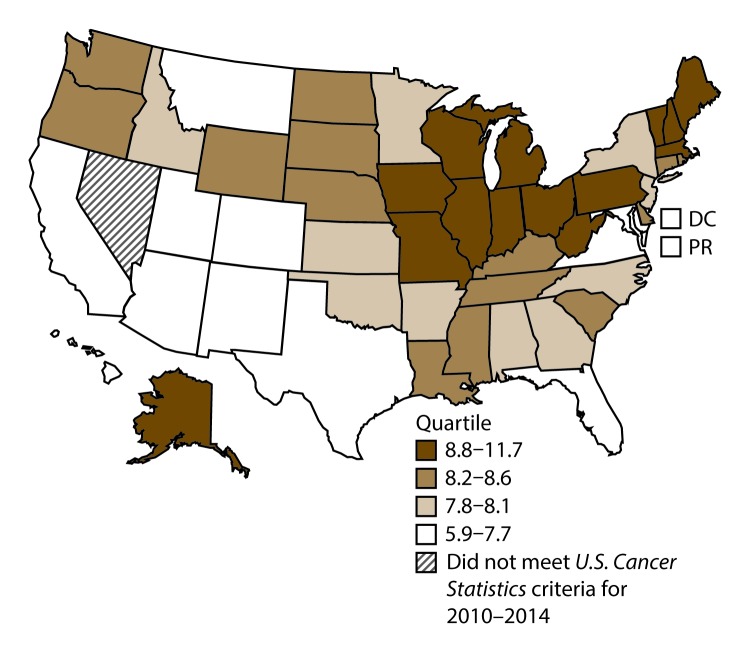
Incidence rates* for male esophageal cancer, by state/ area and U.S. census region^†^ — United States,^§^ 2010–2014 **Abbreviations:** DC = District of Columbia; PR = Puerto Rico. * New cases diagnosed per 100,000 males, age adjusted to the 2000 U.S. standard population. ^^†^^ *West:* 6.9; *Midwest:* 8.9; *Northeast:* 8.6; *South:* 7.7. (*West:* Alaska, Arizona, California, Colorado, Hawaii, Idaho, Montana, Oregon, New Mexico, Utah, Washington, and Wyoming; *Midwest:* Illinois, Indiana, Iowa, Kansas, Michigan, Minnesota, Missouri, Nebraska, North Dakota, Ohio, South Dakota, and Wisconsin; *Northeast:* Connecticut, Maine, Massachusetts, New Hampshire, New Jersey, New York, Pennsylvania, Rhode Island, and Vermont; *South:* Alabama, Arkansas, Delaware, District of Columbia, Florida, Georgia, Kentucky, Louisiana, Maryland, Mississippi, North Carolina, Oklahoma, South Carolina, Tennessee, Texas, Virginia, and West Virginia.) ^§^ Cancer incidence data were compiled from cancer registries that met the data quality criteria for all invasive cancer sites combined, representing approximately 99% of the U.S. population. (Data from Nevada did not meet *U.S. Cancer Statistics* publication criteria for 2010–2014.) Data for Puerto Rico are included in state-specific analyses but not in U.S. census region analyses.

**FIGURE 8:**
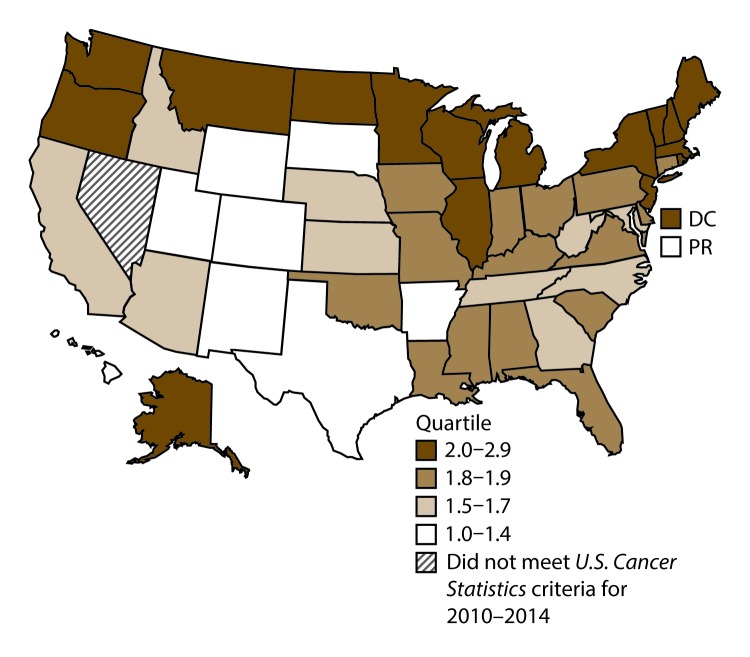
Incidence rates* for female esophageal cancer, by state/ area and U.S. census region^†^ — United States,^§^ 2010–2014 **Abbreviations:** DC = District of Columbia; PR = Puerto Rico. * New cases diagnosed per 100,000 females, age adjusted to the 2000 U.S. standard population. ^†^ *West:* 1.6; *Midwest:* 1.9; *Northeast:* 2.0; *South:* 1.7. (*West:* Alaska, Arizona, California, Colorado, Hawaii, Idaho, Montana, Oregon, New Mexico, Utah, Washington, and Wyoming; *Midwest:* Illinois, Indiana, Iowa, Kansas, Michigan, Minnesota, Missouri, Nebraska, North Dakota, Ohio, South Dakota, and Wisconsin; *Northeast:* Connecticut, Maine, Massachusetts, New Hampshire, New Jersey, New York, Pennsylvania, Rhode Island, and Vermont; *South:* Alabama, Arkansas, Delaware, District of Columbia, Florida, Georgia, Kentucky, Louisiana, Maryland, Mississippi, North Carolina, Oklahoma, South Carolina, Tennessee, Texas, Virginia, and West Virginia.) ^§^ Cancer incidence data were compiled from cancer registries that met the data quality criteria for all invasive cancer sites combined, representing approximately 99% of the U.S. population. (Data from Nevada did not meet *U.S. Cancer Statistics* publication criteria for 2010–2014.) Data for Puerto Rico are included in state-specific analyses but not in U.S. census region analyses.

A total of 81,608 new cases (4.6 per 100,000 persons) of esophageal cancer were reported in the United States during 2010–2014 (Table 4). Incidence rates were higher among men (8.0) than among women (1.8). Rates increased with age and peaked among those aged ≥80 years (45.0 among men and 12.6 among women). Among men, rates were highest among whites (8.2), followed by blacks (7.1), AI/ANs (5.3), and A/PIs (3.7). Among women, rates were highest among blacks (2.3), followed by whites (1.7), AI/ANs (1.4), and A/PIs (1.0). Rates were higher among non-Hispanics (4.8) than Hispanics (2.8). Among those with known tumor characteristics (94.7%), approximately two thirds (65.4%) of all esophageal cancer cases were adenocarcinomas, and slightly less than one third were squamous cell carcinomas (29.6%). This pattern was consistent among men (71.1% adenocarcinomas and 24.2% squamous cell carcinomas). However, the pattern differed among women, who had greater percentages of squamous cell carcinomas (50.1%) than adenocarcinomas (43.9%). More esophageal cancers were diagnosed at regional (32.9%) and distant (36.6%) stages than at the localized stage (19.4%). Men had slightly smaller percentages of localized disease (18.9%) than women (21.6%) and slightly greater percentages of distant disease (38.3%) than women (30.1%). During 2010–2014, esophageal cancer rates were higher in nonmetropolitan counties (5.1) than in metropolitan counties (4.5). Rates among men were highest in the Midwest (8.9) and lowest in the West (6.9). Rates among women were highest in the Northeast and Midwest (1.9–2.0) and lowest in the South and West (1.6–1.7). Vermont, Maine, and New Hampshire had some of the highest rates among men (10.2–11.7) and women (2.3–2.4) (Figures 7 and 8).

**TABLE 4:** Incidence rates* and percentages^†^ of invasive esophageal cancer, by demographic and tumor characteristics — United States,^§^ 2010–2014

Demographic characteristic	Total		Male			Female	
	No.	Rate (95% CI)	No.	Rate	(95% CI)	No.	Rate (95% CI)
**Total**	**81,608**	**4.6 (4.6–4.6)**	**64,311**	**8.0**	**(7.9–8.0)**	**17,297**	**1.8 (1.8–1.8)**
**Age group at diagnosis (yrs)**							
<40	706	0.1 (0.1–0.1)	562	0.2	(0.1–0.2)	144	0.0 (0.0–0.0)
40–49	3,951	1.8 (1.8–1.9)	3,223	3.0	(2.9–3.1)	728	0.7 (0.6–0.7)
50–59	15,943	7.3 (7.1–7.4)	13,075	12.2	(12.0–12.4)	2,868	2.6 (2.5–2.7)
60–69	25,765	16.5 (16.3–16.7)	21,335	28.8	(28.4–29.2)	4,430	5.4 (5.3–5.6)
70–79	20,982	24.0 (23.7–24.3)	16,480	41.9	(41.3–42.6)	4,502	9.4 (9.1–9.7)
≥80	14,261	24.8 (24.4–25.2)	9,636	45.0	(44.1–45.9)	4,625	12.6 (12.3–13.0)
**Race**							
White	71,002	4.7 (4.7–4.8)	56,735	8.2	(8.2–8.3)	14,267	1.7 (1.7–1.8)
Black	7,984	4.3 (4.2–4.4)	5,581	7.1	(6.9–7.3)	2,403	2.3 (2.2–2.4)
American Indian/Alaska Native	460	3.2 (2.8–3.5)	357	5.3	(4.7–5.9)	103	1.4 (1.1–1.7)
Asian/Pacific Islander	1,659	2.2 (2.1–2.3)	1,257	3.7	(3.5–3.9)	402	1.0 (0.9–1.1)
**Ethnicity^¶^**							
Hispanic	4,157	2.8 (2.7–2.9)	3,284	4.9	(4.7–5.1)	873	1.1 (1.0–1.2)
Non-Hispanic	77,446	4.8 (4.7–4.8)	61,025	8.3	(8.2–8.3)	16,421	1.8 (1.8–1.9)
**County classification**							
Metropolitan	64,726	4.5 (4.5–4.5)	50,559	7.8	(7.7–7.9)	14,167	1.8 (1.8–1.8)
Nonmetropolitan	13,056	5.1 (5.0–5.1)	10,693	8.9	(8.7–9.1)	2,363	1.7 (1.6–1.8)
**Census region**							
Northeast	16,751	4.9 (4.9–5.0)	12,913	8.6	(8.5–8.8)	3,838	2.0 (1.9–2.1)
Midwest	20,158	5.1 (5.0–5.2)	15,996	8.9	(8.8–9.1)	4,162	1.9 (1.8–2.0)
South	29,532	4.4 (4.4–4.5)	23,471	7.7	(7.6–7.8)	6,061	1.7 (1.6–1.7)
West	15,167	4.0 (4.0–4.1)	11,931	6.9	(6.8–7.0)	3,236	1.6 (1.6–1.7)
Tumor characteristic**	**No.**	**%**	**No.**	**%**		**No.**	**%**
**Total**	**77,252**	**100.0**	**61,024**	**100.0**		**16,228**	**100.0**
**Histology**							
Squamous cell carcinoma	22,900	29.6	14,768	24.2		8,132	50.1
Adenocarcinoma	50,492	65.4	43,361	71.1		7,131	43.9
Epithelial carcinoma, NOS	2,853	3.7	2,173	3.6		680	4.2
All other histologies	1,007	1.3	722	1.2		285	1.8
**Stage^††^**							
Localized	15,021	19.4	11,522	18.9		3,499	21.6
Regional	25,437	32.9	19,992	32.8		5,445	33.6
Distant	28,248	36.6	23,359	38.3		4,889	30.1
Unknown	8,546	11.1	6,151	10.1		2,395	14.8

**Abbreviations:** CI = confidence interval; NOS = not otherwise specified.

* New cases diagnosed per 100,000 persons, age adjusted to the 2000 U.S. standard population.

^†^ New cases diagnosed.

^§^ Cancer incidence data were compiled from cancer registries that met the data quality criteria for all invasive cancer sites combined, representing approximately 99% of the U.S. population. (Data from Nevada did not meet *U.S. Cancer Statistics* publication criteria for 2010–2014.)

^¶^ Ethnicity is not mutually exclusive from race.

** Only includes microscopically confirmed cases; excludes cases identified only through death certificate or autopsy report.

^††^ Localized: cancer that is confined to the primary site; regional: cancer that has spread directly beyond the primary site or to regional lymph nodes; distant: cancer that has spread to other organs.

### Stomach Cancer

**FIGURE 9:**
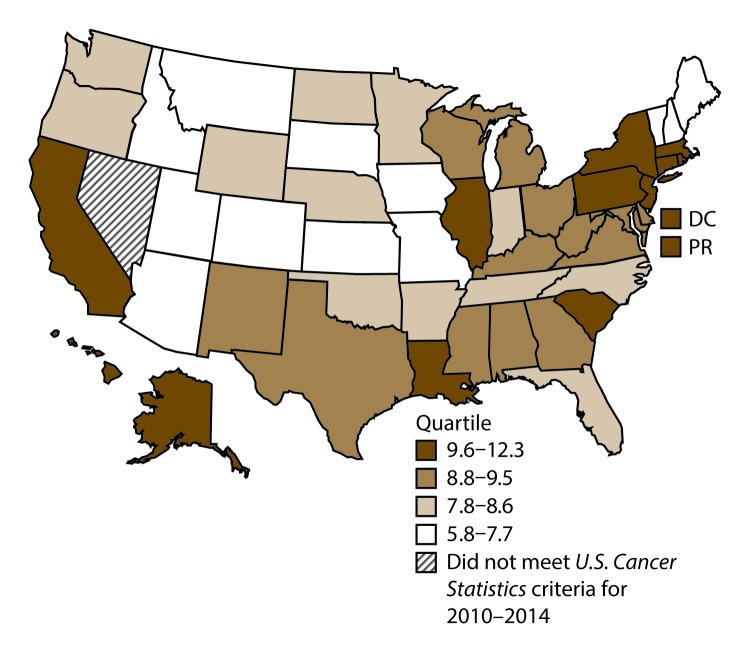
Incidence rates* for male stomach cancer, by state/area and U.S. census region^†^ — United States,^§^ 2010–2014 **Abbreviations:** DC = District of Columbia; PR = Puerto Rico. * New cases diagnosed per 100,000 males, age adjusted to the 2000 U.S. standard population. ^†^ *West:* 9.1; *Midwest:* 8.7; *Northeast:* 10.7; *South:* 8.7. (*West:* Alaska, Arizona, California, Colorado, Hawaii, Idaho, Montana, Oregon, New Mexico, Utah, Washington, and Wyoming; *Midwest:* Illinois, Indiana, Iowa, Kansas, Michigan, Minnesota, Missouri, Nebraska, North Dakota, Ohio, South Dakota, and Wisconsin; *Northeast:* Connecticut, Maine, Massachusetts, New Hampshire, New Jersey, New York, Pennsylvania, Rhode Island, and Vermont; *South:* Alabama, Arkansas, Delaware, District of Columbia, Florida, Georgia, Kentucky, Louisiana, Maryland, Mississippi, North Carolina, Oklahoma, South Carolina, Tennessee, Texas, Virginia, and West Virginia.) ^§^ Cancer incidence data were compiled from cancer registries that met the data quality criteria for all invasive cancer sites combined, representing approximately 99% of the U.S. population. (Data from Nevada did not meet *U.S. Cancer Statistics* publication criteria for 2010–2014.) Data for Puerto Rico are included in state-specific analyses but not in U.S. census region analyses.

**FIGURE 10:**
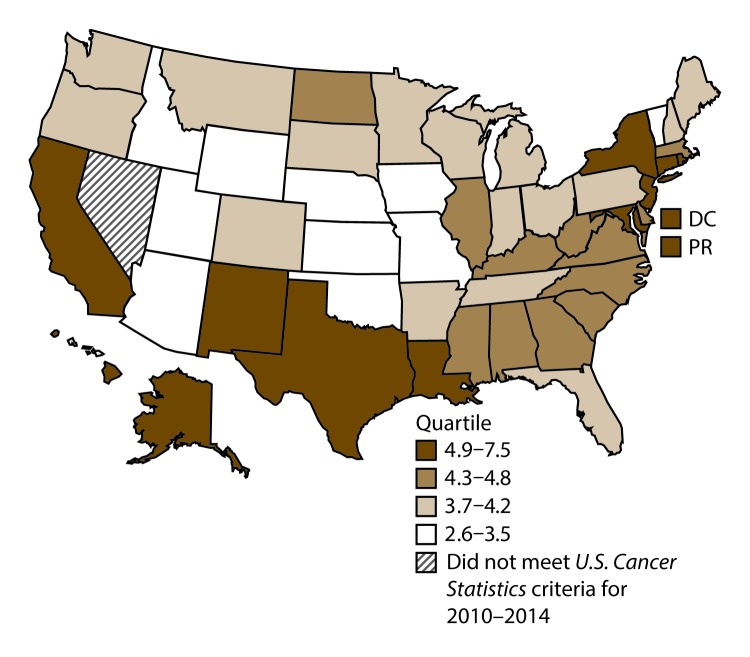
Incidence rates* for female stomach cancer, by state/area and U.S. census region^†^ — United States,^§^ 2010–2014 **Abbreviations:** DC = District of Columbia; PR = Puerto Rico. * New cases diagnosed per 100,000 females, age adjusted to the 2000 U.S. standard population. ^†^ *West:* 4.8; *Midwest:* 4.0; *Northeast:* 5.2; *South:* 4.6 (*West:* Alaska, Arizona, California, Colorado, Hawaii, Idaho, Montana, Oregon, New Mexico, Utah, Washington, and Wyoming; *Midwest:* Illinois, Indiana, Iowa, Kansas, Michigan, Minnesota, Missouri, Nebraska, North Dakota, Ohio, South Dakota, and Wisconsin; *Northeast:* Connecticut, Maine, Massachusetts, New Hampshire, New Jersey, New York, Pennsylvania, Rhode Island, and Vermont; *South:* Alabama, Arkansas, Delaware, District of Columbia, Florida, Georgia, Kentucky, Louisiana, Maryland, Mississippi, North Carolina, Oklahoma, South Carolina, Tennessee, Texas, Virginia, and West Virginia.) ^§^ Cancer incidence data were compiled from cancer registries that met the data quality criteria for all invasive cancer sites combined, representing approximately 99% of the U.S. population. (Data from Nevada did not meet *U.S. Cancer Statistics* publication criteria for 2010–2014.) Data for Puerto Rico are included in state-specific analyses but not in U.S. census region analyses.

A total of 115,147 new cases (6.7 per 100,000 persons) of stomach cancer were reported in the United States during 2010–2014 (Table 5). Incidence rates were higher among men than among women (9.2 and 4.6, respectively). Rates increased with increasing age and peaked among adults aged ≥80 years (43.6). A/PIs had the highest rate (10.7), followed by blacks (10.3), AI/ANs (6.3), and whites (6.0). Hispanics had higher rates than non-Hispanics (9.9 and 6.3, respectively). Among those with known tumor characteristics (96.2%), approximately 86.3% of stomach cancers were adenocarcinomas; gastrointestinal stromal tumors accounted for 8.9%. Approximately 60% of stomach cancers were diagnosed at the regional (26.3%) or distant stage (33.6%), with women having greater percentages of localized disease than men (34.2% and 26.7%, respectively). During 2010–2014, stomach cancer rates were higher in metropolitan counties (6.9) than in nonmetropolitan counties (5.8). Among men, rates were highest in the Northeast (10.7) and lowest in the Midwest and South (8.7). Among women, rates were highest in the Northeast (5.2) and lowest in the Midwest (4.0). DC, Hawaii, New Jersey, New York, Puerto Rico, and Rhode Island had some of the highest rates among men and women (Figures 9 and 10).

**TABLE 5:** Incidence rates* and percentages^†^ of invasive stomach cancer, by demographic and tumor characteristics — United States,^§^ 2010–2014

Demographic characteristic	Total		Male		Female	
	No.	Rate (95% CI)	No.	Rate (95% CI)	No.	Rate (95% CI)
**Total**	**115,147**	**6.7 (6.6–6.7)**	**71,429**	**9.2 (9.1–9.3)**	**43,718**	**4.6 (4.6–4.7)**
**Age group at diagnosis (yrs)**						
<40	3,543	0.5 (0.4–0.5)	1,812	0.5 (0.4–0.5)	1,731	0.4 (0.4–0.5)
40–49	8,145	3.8 (3.7–3.9)	4,733	4.4 (4.3–4.6)	3,412	3.2 (3.1–3.3)
50–59	19,704	9.0 (8.9–9.2)	12,852	12.1 (11.9–12.3)	6,852	6.2 (6.0–6.3)
60–69	29,093	18.7 (18.5–18.9)	19,548	26.4 (26.1–26.8)	9,545	11.7 (11.5–12.0)
70–79	29,473	33.9 (33.5–34.3)	18,938	48.4 (47.7–49.1)	10,535	22.0 (21.6–22.4)
≥80	25,189	43.6 (43.0–44.1)	13,546	63.4 (62.3–64.5)	11,643	31.8 (31.2–32.4)
**Race**						
White	86,976	6.0 (5.9–6.0)	55,378	8.3 (8.3–8.4)	31,598	4.0 (3.9–4.0)
Black	17,936	10.3 (10.1–10.5)	10,169	14.1 (13.8–14.4)	7,767	7.7 (7.6–7.9)
American Indian/Alaska Native	877	6.3 (5.8–6.7)	521	8.3 (7.6–9.2)	356	4.7 (4.2–5.2)
Asian/Pacific Islander	8,011	10.7 (10.4–10.9)	4,596	14.1 (13.6–14.5)	3,415	8.1 (7.8–8.4)
**Ethnicity^¶^**						
Hispanic	15,364	9.9 (9.8–10.1)	8,722	12.8 (12.5–13.1)	6,642	7.7 (7.6–7.9)
Non-Hispanic	99,765	6.3 (6.3–6.4)	62,697	8.8 (8.8–8.9)	37,068	4.3 (4.2–4.3)
**County classification**						
Metropolitan	96,346	6.9 (6.8–6.9)	59,281	9.5 (9.4–9.5)	37,065	4.8 (4.8–4.9)
Nonmetropolitan	14,432	5.8 (5.7–5.9)	9,256	8.0 (7.8–8.2)	5,176	3.9 (3.8–4.0)
**Census region**						
Northeast	25,142	7.6 (7.5–7.7)	15,455	10.7 (10.5–10.8)	9,687	5.2 (5.1–5.3)
Midwest	23,656	6.1 (6.1–6.2)	15,148	8.7 (8.6–8.9)	8,508	4.0 (4.0–4.1)
South	41,502	6.4 (6.4–6.5)	25,497	8.7 (8.6–8.9)	16,005	4.6 (4.5–4.6)
West	24,847	6.8 (6.7–6.9)	15,329	9.1 (9.0–9.3)	9,518	4.8 (4.7–4.9)
Tumor characteristic**	**No.**	**%**	**No.**	**%**	**No.**	**%**
**Total**	**110,731**	**100.0**	**68,956**	**100.0**	**41,775**	**100.0**
**Histology**						
Squamous cell carcinoma	1,091	1.0	772	1.1	319	0.8
Adenocarcinoma	95,611	86.3	60,701	88.0	34,910	83.6
Epithelial carcinoma, NOS	2,881	2.6	1,810	2.6	1,071	2.6
Gastrointestinal stromal tumor	9,819	8.9	4,887	7.1	4,932	11.8
All other histologies	1,329	1.2	786	1.1	543	1.3
**Stage^††^**						
Localized	32,689	29.5	18,397	26.7	14,292	34.2
Regional	29,104	26.3	19,554	28.4	9,550	22.9
Distant	37,226	33.6	24,515	35.6	12,711	30.4
Unknown	11,712	10.6	6,490	9.4	5,222	12.5

**Abbreviations:** CI = confidence interval; NOS = not otherwise specified.

* New cases diagnosed per 100,000 persons, age adjusted to the 2000 U.S. standard population.

^†^ New cases diagnosed.

^§^ Cancer incidence data were compiled from cancer registries that met the data quality criteria for all invasive cancer sites combined, representing approximately 99% of the U.S. population. (Data from Nevada did not meet *U.S. Cancer Statistics* publication criteria for 2010–2014.)

^¶^ Ethnicity is not mutually exclusive from race.

** Only includes microscopically confirmed cases; excludes cases identified only through death certificate or autopsy report.

^††^ Localized: cancer that is confined to the primary site; regional: cancer that has spread directly beyond the primary site or to regional lymph nodes; distant: cancer that has spread to other organs.

### Colon and Rectal Cancers

**FIGURE 11:**
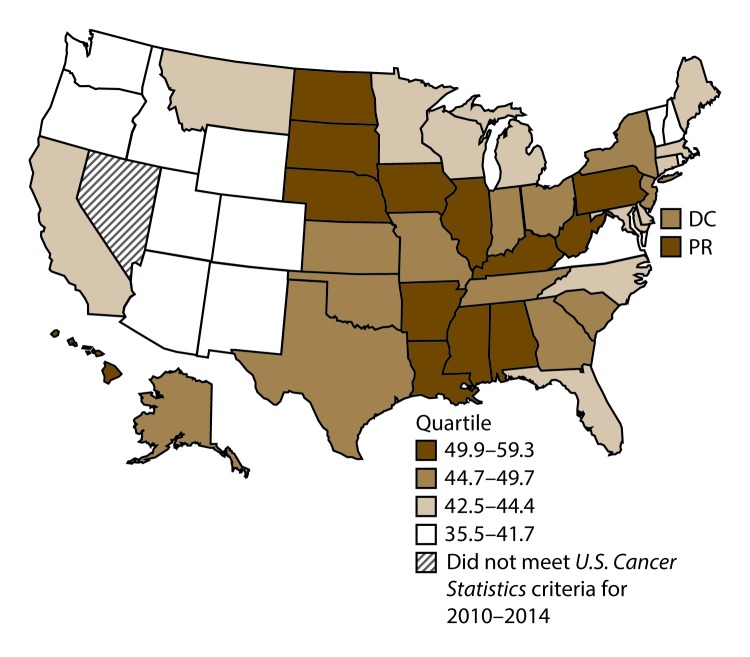
Incidence rates* for male colon and rectal cancers, by state/area and U.S. census region^†^ — United States,^§^ 2010–2014 **Abbreviations:** DC = District of Columbia; PR = Puerto Rico. * New cases diagnosed per 100,000 males, age adjusted to the 2000 U.S. standard population. ^†^ *West:* 41.4; *Midwest:* 48.0; *Northeast:* 46.7; *South:* 46.6. (*West:* Alaska, Arizona, California, Colorado, Hawaii, Idaho, Montana, Oregon, New Mexico, Utah, Washington, and Wyoming; *Midwest:* Illinois, Indiana, Iowa, Kansas, Michigan, Minnesota, Missouri, Nebraska, North Dakota, Ohio, South Dakota, and Wisconsin; *Northeast:* Connecticut, Maine, Massachusetts, New Hampshire, New Jersey, New York, Pennsylvania, Rhode Island, and Vermont; *South:* Alabama, Arkansas, Delaware, District of Columbia, Florida, Georgia, Kentucky, Louisiana, Maryland, Mississippi, North Carolina, Oklahoma, South Carolina, Tennessee, Texas, Virginia, and West Virginia.) ^§^ Cancer incidence data were compiled from cancer registries that met the data quality criteria for all invasive cancer sites combined, representing approximately 99% of the U.S. population. (Data from Nevada did not meet *U.S. Cancer Statistics* publication criteria for 2010–2014.) Data for Puerto Rico are included in state-specific analyses but not in U.S. census region analyses.

**FIGURE 12:**
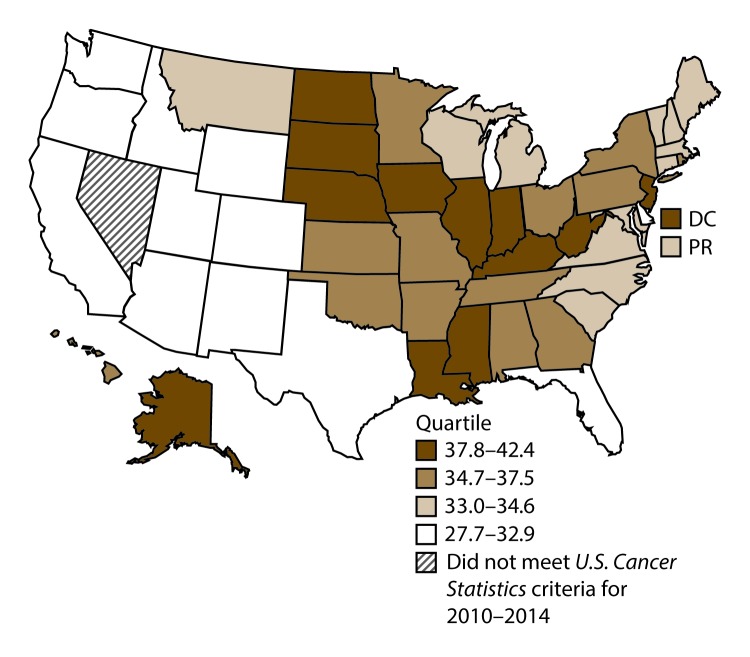
Incidence rates* for female colon and rectal cancers, by state/area and U.S. census region^†^ — United States,^§^ 2010–2014 **Abbreviations:** DC = District of Columbia; PR = Puerto Rico. * New cases diagnosed per 100,000 females, age adjusted to the 2000 U.S. standard population. ^†^ *West:* 32.0; *Midwest:* 36.4; *Northeast:* 36.0; *South:* 34.7. (*West:* Alaska, Arizona, California, Colorado, Hawaii, Idaho, Montana, Oregon, New Mexico, Utah, Washington, and Wyoming; *Midwest:* Illinois, Indiana, Iowa, Kansas, Michigan, Minnesota, Missouri, Nebraska, North Dakota, Ohio, South Dakota, and Wisconsin; *Northeast:* Connecticut, Maine, Massachusetts, New Hampshire, New Jersey, New York, Pennsylvania, Rhode Island, and Vermont; *South:* Alabama, Arkansas, Delaware, District of Columbia, Florida, Georgia, Kentucky, Louisiana, Maryland, Mississippi, North Carolina, Oklahoma, South Carolina, Tennessee, Texas, Virginia, and West Virginia.) ^§^ Cancer incidence data were compiled from cancer registries that met the data quality criteria for all invasive cancer sites combined, representing approximately 99% of the U.S. population. (Data from Nevada did not meet *U.S. Cancer Statistics* publication criteria for 2010–2014.) Data for Puerto Rico are included in state-specific analyses but not in U.S. census region analyses.

A total of 689,738 new cases (39.8 per 100,000 persons) of colon and rectal cancers were reported in the United States during 2010–2014 (Table 6). Incidence rates were substantially higher among men (45.8) than among women (34.8). Rates increased with age and peaked in women and men aged ≥80 years. Rates were highest among blacks (46.7), followed by whites (38.9), A/PIs (31.4), and AI/ANs (30.9). Non-Hispanics had higher rates than Hispanics (40.3 and 35.0, respectively). Among those with known tumor characteristics (96.1%), approximately two thirds (67.9%) of all colon and rectal cancers were adenocarcinomas; adenomas accounted for 17.5%. Approximately 60% of colon and rectal cancers were diagnosed at the regional or distant stage (55.8%), with similar distributions among women and men. During 2010–2014, colon and rectal cancer rates were slightly higher in nonmetropolitan counties (43.1) than in metropolitan counties (39.1). Rates among men and women were highest in the Midwest (48.0 and 36.4, respectively) and lowest in the West (41.4 and 32.0, respectively). Louisiana, Mississippi, and Kentucky had the highest rates both among men (56.0–59.3) and women (41.4–42.4) (Figures 11 and 12).

**TABLE 6:** Incidence rates* and percentages^†^ of invasive colon and rectal cancers, by demographic and tumor characteristics — United States,^§^ 2010–2014

Demographic characteristic	Total		Male		Female	
	No.	Rate (95% CI)	No.	Rate (95% CI)	No.	Rate (95% CI)
**Total**	**689,738**	**39.8 (39.7–39.9)**	**358,980**	**45.8 (45.6–45.9)**	**330,758**	**34.8 (34.7–34.9)**
**Age group at diagnosis (yrs)**						
<40	19,731	2.6 (2.5–2.6)	9,987	2.6 (2.5–2.6)	9,744	2.5 (2.5–2.6)
40–49	52,570	24.3 (24.1–24.5)	28,010	26.1 (25.8–26.5)	24,560	22.5 (22.3–22.8)
50–59	132,893	61.5 (61.2–61.8)	74,955	71.0 (70.5–71.5)	57,938	52.5 (52.0–52.9)
60–69	167,619	107.8 (107.3–108.3)	96,144	130.0 (129.2–130.9)	71,475	87.6 (87.0–88.3)
70–79	162,100	186.3 (185.4–187.3)	85,303	218.0 (216.6–219.5)	76,797	160.5 (159.3–161.6)
≥80	154,825	267.2 (265.9–268.5)	64,581	302.4 (300.0–304.7)	90,244	246.3 (244.7–248.0)
**Race**						
White	568,027	38.9 (38.8–39.0)	297,149	44.7 (44.5–44.8)	270,878	33.9 (33.8–34.1)
Black	84,797	46.7 (46.4–47.1)	42,304	55.1 (54.6–55.7)	42,493	40.9 (40.5–41.3)
American Indian/Alaska Native	4,533	30.9 (29.9–31.9)	2,367	34.7 (33.2–36.3)	2,166	27.8 (26.6–29.0)
Asian/Pacific Islander	24,651	31.4 (31.0–31.8)	12,927	37.0 (36.3–37.6)	11,724	27.0 (26.5–27.5)
**Ethnicity^¶^**						
Hispanic	54,664	35.0 (34.7–35.3)	29,604	42.0 (41.5–42.6)	25,060	29.4 (29.0–29.8)
Non-Hispanic	635,010	40.3 (40.2–40.4)	329,348	46.3 (46.2–46.5)	305,662	35.4 (35.3–35.5)
**County classification**						
Metropolitan	550,090	39.1 (39.0–39.2)	284,709	45.0 (44.8–45.2)	265,381	34.2 (34.1–34.3)
Nonmetropolitan	107,117	43.1 (42.8–43.4)	56,914	49.3 (48.9–49.8)	50,203	37.7 (37.4–38.0)
**Census region**						
Northeast	134,889	40.8 (40.5–41.0)	67,880	46.7 (46.3–47.0)	67,009	36.0 (35.7–36.3)
Midwest	160,438	41.6 (41.4–41.8)	83,150	48.0 (47.6–48.3)	77,288	36.4 (36.1–36.6)
South	259,826	40.0 (39.9–40.2)	137,204	46.6 (46.3–46.8)	122,622	34.7 (34.5–34.9)
West	134,585	36.3 (36.1–36.5)	70,746	41.4 (41.1–41.7)	63,839	32.0 (31.8–32.3)
Tumor characteristic**	**No.**	**%**	**No.**	**%**	**No.**	**%**
**Total**	**663,134**	**100.0**	**346,670**	**100.0**	**316,464**	**100.0**
**Histology**						
Adenocarcinoma	450,086	67.9	237,862	68.6	212,224	67.1
Adenoma	115,940	17.5	62,459	18.0	53,481	16.9
Mucinous carcinoma	50,295	7.6	24,365	7.0	25,930	8.2
Carcinoid	28,410	4.3	13,624	3.9	14,786	4.7
Epithelial carcinoma, NOS	7,140	1.1	3,723	1.1	3,417	1.1
All other histologies	11,263	1.7	4,637	1.3	6,626	2.1
**Stage^††^**						
Localized	260,739	39.3	135,705	39.1	125,034	39.5
Regional	233,918	35.3	121,636	35.1	112,282	35.5
Distant	135,731	20.5	72,393	20.9	63,338	20.0
Unknown	32,746	4.9	16,936	4.9	15,810	5.0

**Abbreviations:** CI = confidence interval; NOS = not otherwise specified.

* New cases diagnosed per 100,000 persons, age adjusted to the 2000 U.S. standard population.

^†^ New cases diagnosed.

^§^ Cancer incidence data were compiled from cancer registries that met the data quality criteria for all invasive cancer sites combined, representing approximately 99% of the U.S. population. (Data from Nevada did not meet *U.S. Cancer Statistics* publication criteria for 2010–2014.)

^¶^ Ethnicity is not mutually exclusive from race.

** Only includes microscopically confirmed cases; excludes cases identified only through death certificate or autopsy report.

^††^ Localized: cancer that is confined to the primary site; regional: cancer that has spread directly beyond the primary site or to regional lymph nodes; distant: cancer that has spread to other organs.

### Liver Cancer

**FIGURE 13:**
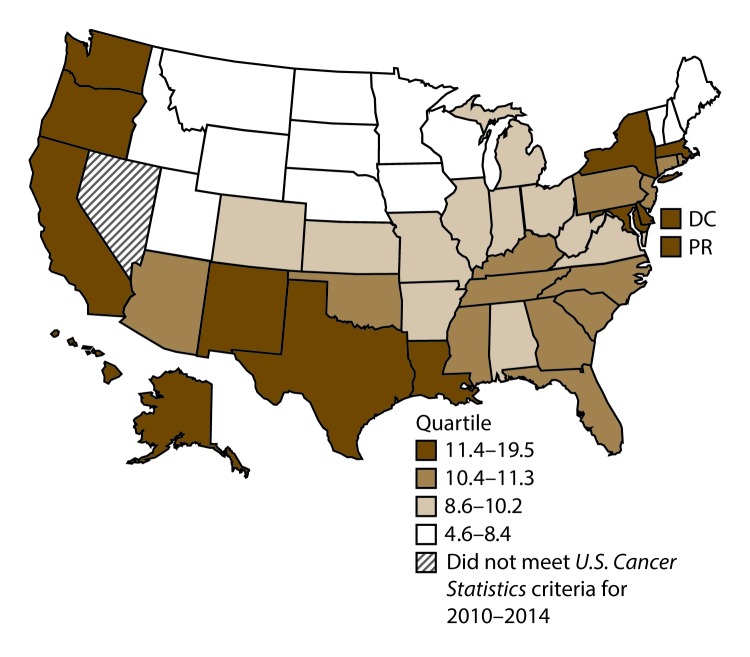
Incidence rates* for male liver cancer, by state/area and U.S. census region^†^ — United States,^§^ 2010–2014 **Abbreviations:** DC = District of Columbia; PR = Puerto Rico. * New cases diagnosed per 100,000 males, age adjusted to the 2000 U.S. standard population. ^†^ *West:* 12.2; *Midwest:* 8.8; *Northeast:* 11.2; *South:* 11.5. (*West:* Alaska, Arizona, California, Colorado, Hawaii, Idaho, Montana, Oregon, New Mexico, Utah, Washington, and Wyoming; *Midwest:* Illinois, Indiana, Iowa, Kansas, Michigan, Minnesota, Missouri, Nebraska, North Dakota, Ohio, South Dakota, and Wisconsin; *Northeast:* Connecticut, Maine, Massachusetts, New Hampshire, New Jersey, New York, Pennsylvania, Rhode Island, and Vermont; *South:* Alabama, Arkansas, Delaware, District of Columbia, Florida, Georgia, Kentucky, Louisiana, Maryland, Mississippi, North Carolina, Oklahoma, South Carolina, Tennessee, Texas, Virginia, and West Virginia.) ^§^ Cancer incidence data were compiled from cancer registries that met the data quality criteria for all invasive cancer sites combined, representing approximately 99% of the U.S. population. (Data from Nevada did not meet *U.S. Cancer Statistics* publication criteria for 2010–2014.) Data for Puerto Rico are included in state-specific analyses but not in U.S. census region analyses.

**FIGURE 14:**
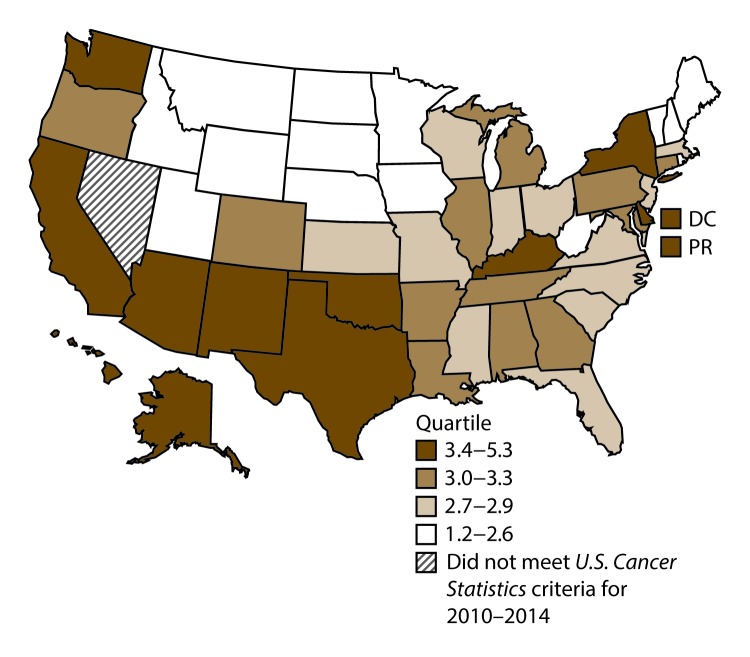
Incidence rates* for female liver cancer, by state/area and U.S. census region^†^ — United States,^§^ 2010–2014 **Abbreviations:** DC = District of Columbia; PR = Puerto Rico. * New cases diagnosed per 100,000 females, age adjusted to the 2000 U.S. standard population. ^†^ *West:* 4.0; *Midwest:* 2.8; *Northeast:* 3.0; *South:* 3.3. (*West:* Alaska, Arizona, California, Colorado, Hawaii, Idaho, Montana, Oregon, New Mexico, Utah, Washington, and Wyoming; *Midwest:* Illinois, Indiana, Iowa, Kansas, Michigan, Minnesota, Missouri, Nebraska, North Dakota, Ohio, South Dakota, and Wisconsin; *Northeast:* Connecticut, Maine, Massachusetts, New Hampshire, New Jersey, New York, Pennsylvania, Rhode Island, and Vermont; *South:* Alabama, Arkansas, Delaware, District of Columbia, Florida, Georgia, Kentucky, Louisiana, Maryland, Mississippi, North Carolina, Oklahoma, South Carolina, Tennessee, Texas, Virginia, and West Virginia.) ^§^ Cancer incidence data were compiled from cancer registries that met the data quality criteria for all invasive cancer sites combined, representing approximately 99% of the U.S. population. (Data from Nevada did not meet *U.S. Cancer Statistics* publication criteria for 2010–2014.) Data for Puerto Rico are included in state-specific analyses but not in U.S. census region analyses.

A total of 126,165 new cases (6.9 per 100,000 persons) of liver cancer were reported in the United States during 2010–2014 (Table 7). Incidence rates were higher among men than among women (11.0 and 3.3, respectively). Among persons aged <80 years, rates increased with increasing age and peaked among adults aged 70–79 years (27.2). Among men, rates were highest among A/PIs (18.5), followed by blacks (16.0), AI/ANs (12.8), and whites (9.8). Among women, rates were highest among A/PIs (6.6), followed by AI/ANs (5.6), blacks (4.3), and whites (2.9). Hispanics had higher rates than non-Hispanics (12.1 and 6.4, respectively). Among those with known tumor characteristics (94.2%), 86.4% of cases of liver cancer were hepatocellular carcinoma, and 7.2% were adenocarcinoma. Men had greater percentages of hepatocellular carcinomas than women (89.1% and 78.3%, respectively), whereas women had greater percentages of adenocarcinomas than men (12.4% and 5.5%, respectively). The majority of liver cancer cases (44.8%) were diagnosed at the localized stage. Women had greater percentages of localized stage liver cancer diagnoses than men (47.5% and 43.9%, respectively), whereas men had greater percentages of regional stage liver cancer diagnoses (28.0% and 24.2%, respectively). During 2010–2014, liver cancer rates were higher in metropolitan counties (7.2) than in nonmetropolitan counties (5.8). Among men and women, liver cancer cases were highest in the West (12.2 and 4.0, respectively) and lowest in the Midwest (8.8 and 2.8, respectively). California, DC, Hawaii, New Mexico, and Texas had some of the highest rates of liver cancer among men (13.6–19.5) and women (3.9–5.3) (Figures 13 and 14).

**TABLE 7:** Incidence rates* and percentages^†^ of invasive liver cancer, by demographic and tumor characteristics — United States,^§^ 2010–2014

Demographic characteristic	Total		Male		Female	
	No.	Rate (95% CI)	No.	Rate (95% CI)	No.	Rate (95% CI)
**Total**	**126,165**	**6.9 (6.8–6.9)**	**94,425**	**11.0 (10.9–11.0)**	**31,740**	**3.3 (3.2–3.3)**
**Age group at diagnosis (yrs)**						
<40	2,589	0.3 (0.3–0.3)	1,609	0.4 (0.4–0.4)	980	0.2 (0.2–0.3)
40–49	5,965	2.7 (2.6–2.8)	4,660	4.3 (4.1–4.4)	1,305	1.2 (1.1–1.3)
50–59	37,661	17.0 (16.9–17.2)	30,832	28.6 (28.3–29.0)	6,829	6.0 (5.9–6.2)
60–69	41,278	26.2 (25.9–26.4)	32,632	43.3 (42.9–43.8)	8,646	10.6 (10.3–10.8)
70–79	23,722	27.2 (26.8–27.5)	16,095	40.9 (40.3–41.5)	7,627	15.9 (15.6–16.3)
≥80	14,950	26.1 (25.7–26.5)	8,597	40.1 (39.3–41.0)	6,353	17.6 (17.1–18.0)
**Race**						
White	94,355	6.2 (6.1–6.2)	70,871	9.8 (9.7–9.9)	23,484	2.9 (2.9–3.0)
Black	19,566	9.5 (9.3–9.6)	14,820	16.0 (15.7–16.3)	4,746	4.3 (4.2–4.4)
American Indian/Alaska Native	1,463	9.0 (8.5–9.6)	1,017	12.8 (12.0–13.7)	446	5.6 (5.1–6.2)
Asian/Pacific Islander	9,463	11.9 (11.7–12.2)	6,734	18.5 (18.1–19.0)	2,729	6.6 (6.3–6.8)
**Ethnicity^¶^**						
Hispanic	19,644	12.1 (11.9–12.3)	14,334	18.7 (18.4–19.0)	5,310	6.5 (6.3–6.7)
Non-Hispanic	106,512	6.4 (6.4–6.4)	80,084	10.3 (10.2–10.3)	26,428	3.0 (3.0–3.0)
**County classification**						
Metropolitan	106,798	7.2 (7.1–7.2)	79,865	11.5 (11.4–11.6)	26,933	3.4 (3.4–3.5)
Nonmetropolitan	15,220	5.8 (5.7–5.9)	11,479	9.2 (9.0–9.4)	3,741	2.8 (2.7–2.9)
**Census region**						
Northeast	23,350	6.8 (6.7–6.8)	17,752	11.2 (11.0–11.3)	5,598	3.0 (2.9–3.1)
Midwest	22,601	5.6 (5.5–5.6)	16,698	8.8 (8.6–8.9)	5,903	2.8 (2.7–2.8)
South	49,130	7.1 (7.1–7.2)	37,028	11.5 (11.4–11.6)	12,102	3.3 (3.3–3.4)
West	31,084	7.9 (7.8–8.0)	22,947	12.2 (12.1–12.4)	8,137	4.0 (3.9–4.1)
Tumor characteristic**	**No.**	**%**	**No.**	**%**	**No.**	**%**
**Total**	**118,896**	**100.0**	**89,081**	**100.0**	**29,815**	**100.0**
**Histology**						
Hepatocellular carcinoma	102,719	86.4	79,381	89.1	23,338	78.3
Adenocarcinoma	8,584	7.2	4,890	5.5	3,694	12.4
Epithelial carcinoma, NOS	1,997	1.7	1,319	1.5	678	2.3
All other histologies	5,596	4.7	3,491	3.9	2,105	7.1
**Stage^††^**						
Localized	53,260	44.8	39,105	43.9	14,155	47.5
Regional	32,202	27.1	24,977	28.0	7,225	24.2
Distant	18,662	15.7	14,421	16.2	4,241	14.2
Unknown	14,772	12.4	10,578	11.9	4,194	14.1

**Abbreviations:** CI = confidence interval; NOS = not otherwise specified.

* New cases diagnosed per 100,000 persons, age adjusted to the 2000 U.S. standard population.

^†^ New cases diagnosed.

^§^ Cancer incidence data were compiled from cancer registries that met the data quality criteria for all invasive cancer sites combined, representing approximately 99% of the U.S. population. (Data from Nevada did not meet *U.S. Cancer Statistics* publication criteria for 2010–2014.)

^¶^ Ethnicity is not mutually exclusive from race.

** Excludes cases identified only through death certificate or autopsy reports.

^††^ Localized: cancer that is confined to the primary site; regional: cancer that has spread directly beyond the primary site or to regional lymph nodes; distant: cancer that has spread to other organs.

### Pancreatic Cancer

**FIGURE 15:**
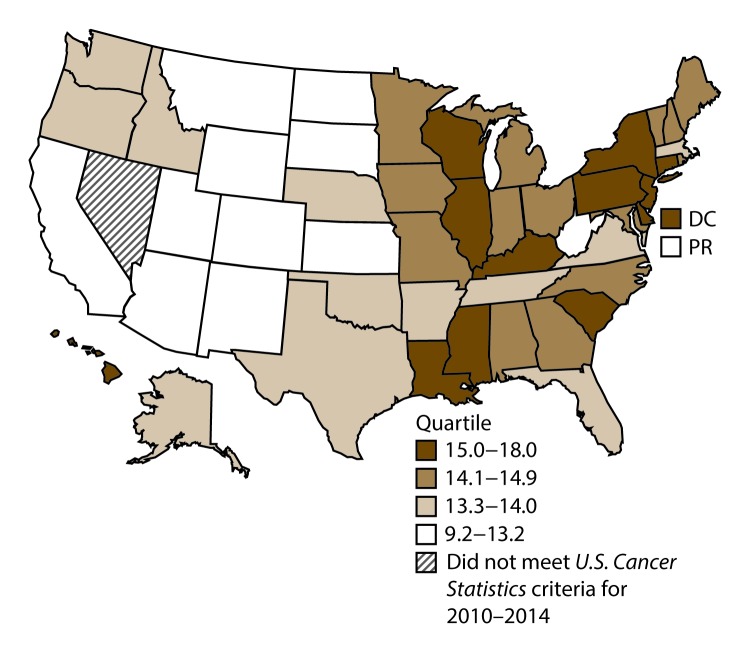
Incidence rates* for male pancreatic cancer, by state/ area and U.S. census region^†^ — United States,^§^ 2010–2014 **Abbreviations:** DC = District of Columbia; PR = Puerto Rico. * New cases diagnosed per 100,000 males, age adjusted to the 2000 U.S. standard population. ^†^ *West:* 13.2; *Midwest:* 14.4; *Northeast:* 15.4; *South:* 14.1. (*West:* Alaska, Arizona, California, Colorado, Hawaii, Idaho, Montana, Oregon, New Mexico, Utah, Washington, and Wyoming; *Midwest:* Illinois, Indiana, Iowa, Kansas, Michigan, Minnesota, Missouri, Nebraska, North Dakota, Ohio, South Dakota, and Wisconsin; *Northeast:* Connecticut, Maine, Massachusetts, New Hampshire, New Jersey, New York, Pennsylvania, Rhode Island, and Vermont; *South:* Alabama, Arkansas, Delaware, District of Columbia, Florida, Georgia, Kentucky, Louisiana, Maryland, Mississippi, North Carolina, Oklahoma, South Carolina, Tennessee, Texas, Virginia, and West Virginia.) ^§^ Cancer incidence data were compiled from cancer registries that met the data quality criteria for all invasive cancer sites combined, representing approximately 99% of the U.S. population. (Data from Nevada did not meet *U.S. Cancer Statistics* publication criteria for 2010–2014.) Data for Puerto Rico are included in state-specific analyses but not in U.S. census region analyses.

**FIGURE 16:**
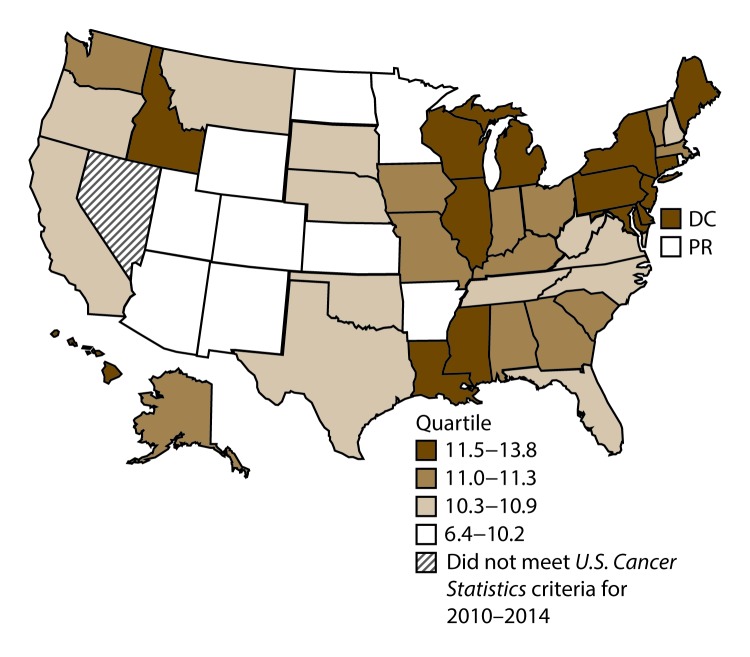
Incidence rates* for female pancreatic cancer, by state/ area and U.S. census region^†^ — United States,^§^ 2010–2014 **Abbreviations:** DC = District of Columbia; PR = Puerto Rico. * New cases diagnosed per 100,000 females, age adjusted to the 2000 U.S. standard population. ^†^ *West:* 10.4; *Midwest:* 11.1; *Northeast:* 12.0; *South:* 10.9. (*West:* Alaska, Arizona, California, Colorado, Hawaii, Idaho, Montana, Oregon, New Mexico, Utah, Washington, and Wyoming; *Midwest:* Illinois, Indiana, Iowa, Kansas, Michigan, Minnesota, Missouri, Nebraska, North Dakota, Ohio, South Dakota, and Wisconsin; *Northeast:* Connecticut, Maine, Massachusetts, New Hampshire, New Jersey, New York, Pennsylvania, Rhode Island, and Vermont; *South:* Alabama, Arkansas, Delaware, District of Columbia, Florida, Georgia, Kentucky, Louisiana, Maryland, Mississippi, North Carolina, Oklahoma, South Carolina, Tennessee, Texas, Virginia, and West Virginia.) ^§^ Cancer incidence data were compiled from cancer registries that met the data quality criteria for all invasive cancer sites combined, representing approximately 99% of the U.S. population. (Data from Nevada did not meet *U.S. Cancer Statistics* publication criteria for 2010–2014.) Data for Puerto Rico are included in state-specific analyses but not in U.S. census region analyses.

A total of 218,919 new cases (12.5 per 100,000 persons) of pancreatic cancer were reported in the United States during 2010–2014 (Table 8). Incidence rates were higher among men than among women (14.2 and 11.0, respectively). Rates increased with increasing age and peaked among adults aged ≥80 years (93.1). Blacks had the highest rates (15.5), followed by whites (12.3), A/PIs (9.3), and AI/ANs (8.2). Non-Hispanics had higher rates than Hispanics (12.6 and 11.1, respectively). Among those with known tumor characteristics (81.9%), 79.7% of pancreatic cancer cases were adenocarcinomas, and 3.3% were mucinous adenocarcinomas. Ductal carcinomas accounted for 9.8% of pancreatic cancer diagnoses. Approximately half (51.5%) of pancreatic cancer cases were diagnosed at the distant stage; 33.8% of pancreatic cancers were diagnosed at the regional stage, and 10.5% were diagnosed at the localized stage. During 2010–2014, pancreatic cancer rates were slightly higher in metropolitan counties (12.6) than in nonmetropolitan counties (12.1). Among men and women, pancreatic cancer rates were highest in the Northeast (15.4 and 12.0, respectively) and lowest in the West (13.2 and 10.4, respectively). DC, Delaware, Hawaii, Louisiana, Mississippi, New Jersey, New York, and Pennsylvania had some of the highest rates of pancreatic cancer among men (15.4–18.0) and women (11.9–13.8) (Figures 15 and 16).

**TABLE 8:** Incidence rates* and percentages^†^ of invasive pancreatic cancer, by demographic and tumor characteristics — United States,^§^ 2010–2014

Demographic characteristic	Total		Male		Female	
	No.	Rate (95% CI)	No.	Rate (95% CI)	No.	Rate (95% CI)
**Total**	**218,919**	**12.5 (12.4–12.6)**	**111,730**	**14.2 (14.1–14.3)**	**107,189**	**11.0 (11.0–11.1)**
**Age group at diagnosis (yrs)**						
<40	2,502	0.3 (0.3–0.3)	1,182	0.3 (0.3–0.3)	1,320	0.3 (0.3–0.4)
40–49	9,445	4.3 (4.2–4.4)	5,345	5.0 (4.8–5.1)	4,100	3.7 (3.6–3.8)
50–59	34,179	15.6 (15.4–15.7)	19,892	18.6 (18.3–18.8)	14,287	12.7 (12.5–12.9)
60–69	59,397	38.2 (37.9–38.6)	33,373	45.2 (44.7–45.7)	26,024	32.0 (31.6–32.4)
70–79	59,506	68.4 (67.8–68.9)	30,244	77.3 (76.4–78.1)	29,262	61.1 (60.4–61.8)
≥80	53,890	93.1 (92.3–93.9)	21,694	101.5 (100.2–102.9)	32,196	87.9 (86.9–88.9)
**Race**						
White	182,531	12.3 (12.2–12.3)	94,557	14.1 (14.0–14.2)	87,974	10.7 (10.6–10.8)
Black	27,174	15.5 (15.3–15.7)	12,671	16.9 (16.6–17.2)	14,503	14.4 (14.2–14.6)
American Indian/Alaska Native	1,131	8.2 (7.7–8.7)	587	9.1 (8.3–10.0)	544	7.4 (6.7–8.0)
Asian/Pacific Islander	6,787	9.3 (9.1–9.5)	3,227	10.0 (9.6–10.4)	3,560	8.7 (8.4–9.0)
**Ethnicity^¶^**						
Hispanic	16,048	11.1 (10.9–11.3)	7,854	12.0 (11.7–12.3)	8,194	10.3 (10.1–10.6)
Non-Hispanic	202,859	12.6 (12.6–12.7)	103,870	14.5 (14.4–14.5)	98,989	11.1 (11.0–11.2)
**County classification**						
Metropolitan	178,483	12.6 (12.5–12.7)	90,388	14.3 (14.2–14.4)	88,095	11.2 (11.1–11.2)
Nonmetropolitan	30,997	12.1 (12.0–12.3)	16,246	13.9 (13.6–14.1)	14,751	10.6 (10.4–10.8)
**Census region**						
Northeast	45,414	13.5 (13.4–13.6)	22,564	15.4 (15.2–15.6)	22,850	12.0 (11.8–12.1)
Midwest	49,381	12.6 (12.5–12.7)	25,272	14.4 (14.3–14.6)	24,109	11.1 (11.0–11.2)
South	80,688	12.4 (12.3–12.4)	41,527	14.1 (14.0–14.3)	39,161	10.9 (10.7–11.0)
West	43,436	11.7 (11.6–11.8)	22,367	13.2 (13.0–13.4)	21,069	10.4 (10.3–10.6)
Tumor characteristic**	**No.**	**%**	**No.**	**%**	**No.**	**%**
**Total**	**179,360**	**100.0**	**93,822**	**100.0**	**85,538**	**100.0**
**Histology**						
Mucinous adenocarcinoma	5,874	3.3	2,943	3.1	2,931	3.4
Other adenocarcinomas	142,872	79.7	74,884	79.8	67,988	79.5
Epithelial carcinoma, NOS	8,105	4.5	4,427	4.7	3,678	4.3
Ductal carcinoma	17,655	9.8	8,999	9.6	8,656	10.1
All other histologies	4,854	2.7	2,569	2.7	2,285	2.7
**Stage^††^**						
Localized	18,904	10.5	9,114	9.7	9,790	11.4
Regional	60,545	33.8	30,867	32.9	29,678	34.7
Distant	92,435	51.5	50,179	53.5	42,256	49.4
Unknown	7,476	4.2	3,662	3.9	3,814	4.5

**Abbreviations:** CI = confidence interval; NOS = not otherwise specified.

* New cases diagnosed per 100,000 persons, age adjusted to the 2000 U.S. standard population.

^†^ New cases diagnosed.

^§^ Cancer incidence data were compiled from cancer registries that met the data quality criteria for all invasive cancer sites combined, representing approximately 99% of the U.S. population. (Data from Nevada did not meet *U.S. Cancer Statistics* publication criteria for 2010–2014.)

^¶^ Ethnicity is not mutually exclusive from race.

** Only includes microscopically confirmed cases; excludes cases identified only through death certificate or autopsy report.

^††^ Localized: cancer that is confined to the primary site; regional: cancer that has spread directly beyond the primary site or to regional lymph nodes; distant: cancer that has spread to other organs.

### Kidney and Renal Pelvis Cancers

**FIGURE 17:**
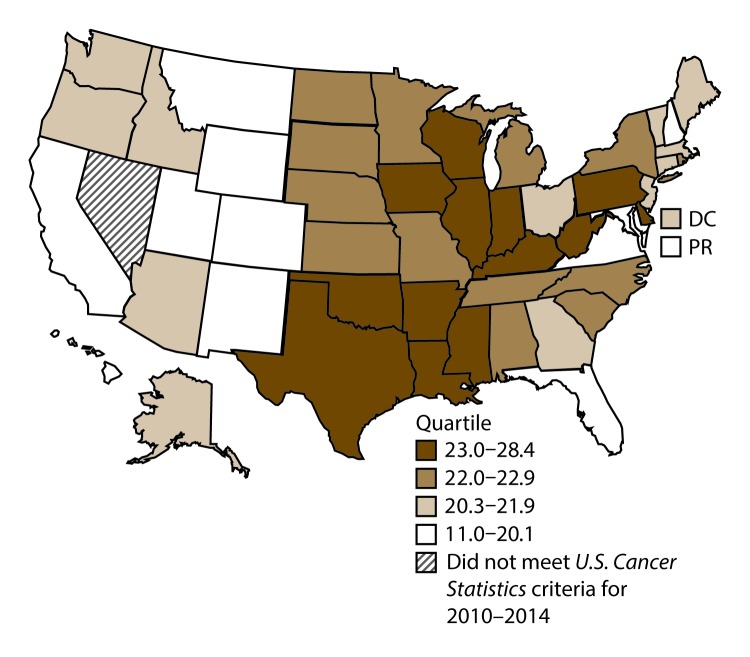
Incidence rates* for male kidney and renal pelvis cancers, by state/area and U.S. census region^†^ — United States,^§^ 2010–2014 **Abbreviations:** DC = District of Columbia; PR = Puerto Rico. * New cases diagnosed per 100,000 males, age adjusted to the 2000 U.S. standard population. ^†^ *West:* 19.7; *Midwest:* 22.9; *Northeast:* 22.3; *South:* 22.2*.* (*West:* Alaska, Arizona, California, Colorado, Hawaii, Idaho, Montana, Oregon, New Mexico, Utah, Washington, and Wyoming; *Midwest:* Illinois, Indiana, Iowa, Kansas, Michigan, Minnesota, Missouri, Nebraska, North Dakota, Ohio, South Dakota, and Wisconsin; *Northeast:* Connecticut, Maine, Massachusetts, New Hampshire, New Jersey, New York, Pennsylvania, Rhode Island, and Vermont; *South:* Alabama, Arkansas, Delaware, District of Columbia, Florida, Georgia, Kentucky, Louisiana, Maryland, Mississippi, North Carolina, Oklahoma, South Carolina, Tennessee, Texas, Virginia, and West Virginia.) ^§^ Cancer incidence data were compiled from cancer registries that met the data quality criteria for all invasive cancer sites combined, representing approximately 99% of the U.S. population. (Data from Nevada did not meet *U.S. Cancer Statistics* publication criteria for 2010–2014.) Data for Puerto Rico are included in state-specific analyses but not in U.S. census region analyses.

**FIGURE 18:**
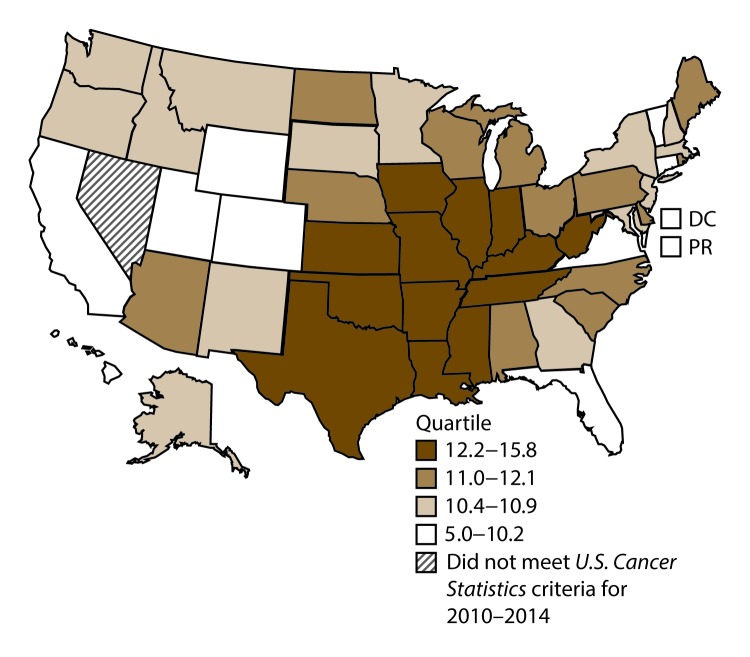
Incidence rates* for female kidney and renal pelvis cancers, by state/area and U.S. census region^†^ — United States,^§^ 2010–2014 **Abbreviations:** DC = District of Columbia; PR = Puerto Rico. * New cases diagnosed per 100,000 females, age adjusted to the 2000 U.S. standard population. ^†^ *West:* 9.9; *Midwest:* 12.1; *Northeast:* 10.9; *South:* 11.8. (*West:* Alaska, Arizona, California, Colorado, Hawaii, Idaho, Montana, Oregon, New Mexico, Utah, Washington, and Wyoming; *Midwest:* Illinois, Indiana, Iowa, Kansas, Michigan, Minnesota, Missouri, Nebraska, North Dakota, Ohio, South Dakota, and Wisconsin; *Northeast:* Connecticut, Maine, Massachusetts, New Hampshire, New Jersey, New York, Pennsylvania, Rhode Island, and Vermont; *South:* Alabama, Arkansas, Delaware, District of Columbia, Florida, Georgia, Kentucky, Louisiana, Maryland, Mississippi, North Carolina, Oklahoma, South Carolina, Tennessee, Texas, Virginia, and West Virginia.) ^§^ Cancer incidence data were compiled from cancer registries that met the data quality criteria for all invasive cancer sites combined, representing approximately 99% of the U.S. population. (Data from Nevada did not meet *U.S. Cancer Statistics* publication criteria for 2010–2014.) Data for Puerto Rico are included in state-specific analyses but not in U.S. census region analyses.

A total of 280,883 new cases (16.1 per 100,000 persons) of kidney and renal pelvis cancers were reported in the United States during 2010–2014 (Table 9). Incidence rates were almost twice as high among men (21.8) than among women (11.3). In persons aged <80 years, rates increased with increasing age and peaked among men and women aged 70–79 years (97.5 and 48.5, respectively). Blacks had the highest rates (17.6), followed by whites (16.3), AI/ANs (15.7), and A/PIs (7.5). Rates were similar among non-Hispanics (16.2) and Hispanics (16.0). Among those with known tumor characteristics (89.7%), the majority (87.9%) of cases of kidney cancer were renal cell carcinomas. Transitional cell carcinoma accounted for 6.7% of cases, and other adenocarcinomas accounted for 2.5% of cases. At diagnosis, 67.2% of cases of kidney cancer were at a localized stage. During 2010–2014, kidney cancer rates were higher in nonmetropolitan counties (17.1) than in metropolitan counties (15.9). Among men, kidney cancer incidence rates were highest in the South, Northeast, and Midwest (22.2–22.9) and lowest in the West (19.7). Among women, rates were highest in the Midwest (12.1) and lowest in the West (9.9). Kentucky, Louisiana, and Mississippi had some of the highest rates of kidney cancer among men (26.2–28.4) and women (14.4–15.8) (Figures 17 and 18).

**TABLE 9:** Incidence rates* and percentages^†^ of invasive kidney and renal pelvis cancers, by demographic and tumor characteristics — United States,^§^ 2010–2014

Demographic characteristic	Total		Male		Female	
	No.	Rate (95% CI)	No.	Rate (95% CI)	No.	Rate (95% CI)
**Total**	**280,883**	**16.1 (16.1–16.2)**	**176,108**	**21.8 (21.7–21.9)**	**104,775**	**11.3 (11.2–11.4)**
**Age group at diagnosis (yrs)**						
<40	13,898	1.8 (1.8–1.8)	7,650	2.0 (1.9–2.0)	6,248	1.6 (1.6–1.7)
40–49	27,897	13.0 (12.8–13.1)	17,920	16.8 (16.5–17.0)	9,977	9.2 (9.0–9.4)
50–59	61,403	28.2 (28.0–28.4)	40,198	37.9 (37.5–38.2)	21,205	19.0 (18.7–19.3)
60–69	81,318	52.2 (51.9–52.6)	53,099	71.8 (71.1–72.4)	28,219	34.6 (34.2–35.0)
70–79	61,763	70.5 (70.0–71.1)	38,439	97.5 (96.5–98.5)	23,324	48.5 (47.9–49.1)
≥80	34,604	60.5 (59.9–61.1)	18,802	87.8 (86.5–89.1)	15,802	44.1 (43.4–44.8)
**Race**						
White	236,281	16.3 (16.2–16.3)	148,897	21.9 (21.8–22.0)	87,384	11.4 (11.3–11.5)
Black	33,396	17.6 (17.4–17.8)	20,108	24.2 (23.8–24.5)	13,288	12.6 (12.4–12.8)
American Indian/Alaska Native	2,526	15.7 (15.1–16.4)	1,510	20.1 (19.0–21.3)	1,016	11.9 (11.2–12.7)
Asian/Pacific Islander	6,057	7.5 (7.3–7.7)	3,926	10.9 (10.5–11.2)	2,131	4.8 (4.6–5.1)
**Ethnicity^¶^**						
Hispanic	27,057	16.0 (15.8–16.2)	16,067	20.8 (20.4–21.1)	10,990	12.1 (11.8–12.3)
Non-Hispanic	253,801	16.2 (16.1–16.3)	160,031	22.0 (21.9–22.1)	93,770	11.2 (11.2–11.3)
**County classification**						
Metropolitan	226,032	15.9 (15.9–16.0)	141,835	21.7 (21.6–21.8)	84,197	11.1 (11.0–11.2)
Nonmetropolitan	41,830	17.1 (16.9–17.2)	25,957	22.2 (21.9–22.5)	15,873	12.5 (12.3–12.7)
**Census region**						
Northeast	52,819	16.1 (16.0–16.3)	33,417	22.3 (22.1–22.6)	19,402	10.9 (10.8–11.1)
Midwest	65,551	17.1 (16.9–17.2)	40,907	22.9 (22.7–23.1)	24,644	12.1 (11.9–12.2)
South	108,341	16.6 (16.5–16.7)	67,177	22.2 (22.1–22.4)	41,164	11.8 (11.7–11.9)
West	54,172	14.5 (14.3–14.6)	34,607	19.7 (19.5–19.9)	19,565	9.9 (9.8–10.1)
Tumor characteristic**	**No.**	**%**	**No.**	**%**	**No.**	**%**
**Total**	**251,844**	**100.0**	**158,949**	**100.0**	**92,895**	**100.0**
**Histology**						
Renal cell carcinoma	221,417	87.9	140,989	88.7	80,428	86.6
Other adenocarcinomas	6,286	2.5	4,060	2.6	2,226	2.4
Transitional cell carcinoma	16,767	6.7	9,805	6.2	6,962	7.5
Epithelial carcinoma, NOS	1,942	0.8	1,217	0.8	725	0.8
All other histologies	5,432	2.2	2,878	1.8	2,554	2.7
**Stage^††^**						
Localized	169,202	67.2	104,164	65.5	65,038	70.0
Regional	42,681	16.9	28,202	17.7	14,479	15.6
Distant	34,681	13.8	23,306	14.7	11,375	12.2
Unknown	5,280	2.1	3,277	2.1	2,003	2.2

**Abbreviations:** CI = confidence interval; NOS = not otherwise specified.

* New cases diagnosed per 100,000 persons, age adjusted to the 2000 U.S. standard population.

^†^ New cases diagnosed.

^§^ Cancer incidence data were compiled from cancer registries that met the data quality criteria for all invasive cancer sites combined, representing approximately 99% of the U.S. population. (Data from Nevada did not meet *U.S. Cancer Statistics* publication criteria for 2010–2014.)

^¶^ Ethnicity is not mutually exclusive from race.

** Only includes microscopically confirmed cases; excludes cases identified only through death certificate or autopsy report.

^††^ Localized: cancer that is confined to the primary site; regional: cancer that has spread directly beyond the primary site or to regional lymph nodes; distant: cancer that has spread to other organs.

### Urinary Bladder Cancer

**FIGURE 19:**
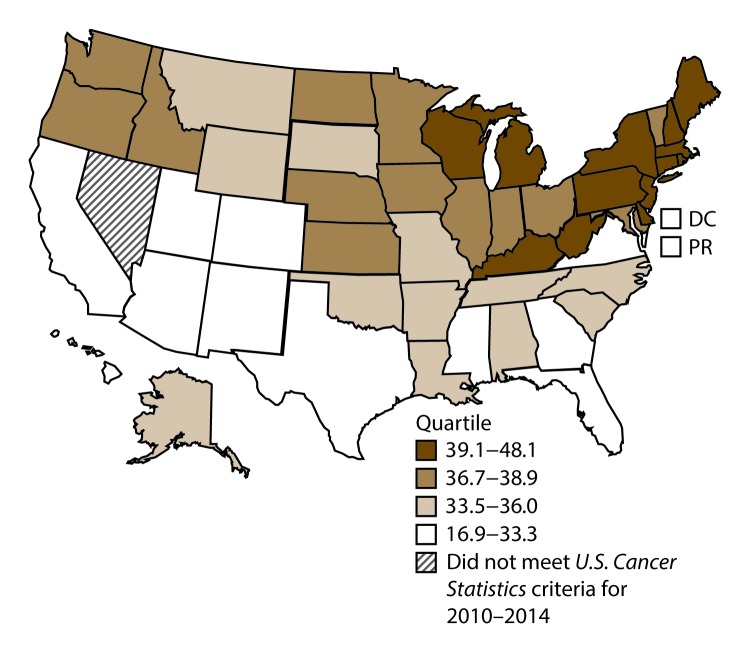
Incidence rates* for male urinary bladder cancer, by state/area and U.S. census region^†^ — United States,^§^ 2010–2014 **Abbreviations:** DC = District of Columbia; PR = Puerto Rico. * New cases diagnosed per 100,000 males, age adjusted to the 2000 U.S. standard population. ^†^ *West:* 32.5; *Midwest:* 37.9; *Northeast:* 42.5; *South:* 33.0. (*West:* Alaska, Arizona, California, Colorado, Hawaii, Idaho, Montana, Oregon, New Mexico, Utah, Washington, and Wyoming; *Midwest:* Illinois, Indiana, Iowa, Kansas, Michigan, Minnesota, Missouri, Nebraska, North Dakota, Ohio, South Dakota, and Wisconsin; *Northeast:* Connecticut, Maine, Massachusetts, New Hampshire, New Jersey, New York, Pennsylvania, Rhode Island, and Vermont; *South:* Alabama, Arkansas, Delaware, District of Columbia, Florida, Georgia, Kentucky, Louisiana, Maryland, Mississippi, North Carolina, Oklahoma, South Carolina, Tennessee, Texas, Virginia, and West Virginia.) ^§^ Cancer incidence data were compiled from cancer registries that met the data quality criteria for all invasive cancer sites combined, representing approximately 99% of the U.S. population. (Data from Nevada did not meet *U.S. Cancer Statistics* publication criteria for 2010–2014.) Data for Puerto Rico are included in state-specific analyses but not in U.S. census region analyses.

**FIGURE 20:**
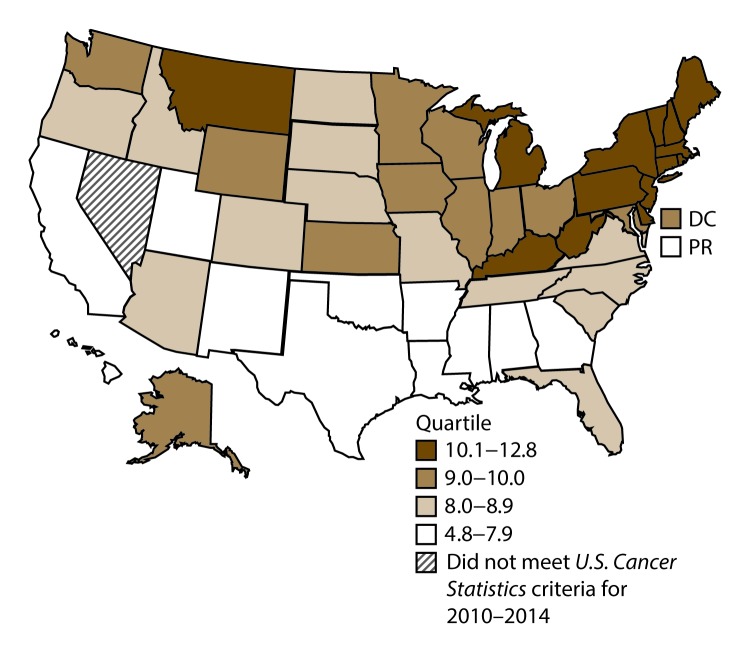
Incidence rates* for female urinary bladder cancer, by state/area and U.S. census region^†^ — United States,^§^ 2010–2014 **Abbreviations:** DC = District of Columbia; PR = Puerto Rico. * New cases diagnosed per 100,000 females, age adjusted to the 2000 U.S. standard population. ^†^ *West:* 7.8; *Midwest:* 9.4; *Northeast:* 11.0; *South:* 8.0. (*West:* Alaska, Arizona, California, Colorado, Hawaii, Idaho, Montana, Oregon, New Mexico, Utah, Washington, and Wyoming; *Midwest:* Illinois, Indiana, Iowa, Kansas, Michigan, Minnesota, Missouri, Nebraska, North Dakota, Ohio, South Dakota, and Wisconsin; *Northeast:* Connecticut, Maine, Massachusetts, New Hampshire, New Jersey, New York, Pennsylvania, Rhode Island, and Vermont; *South:* Alabama, Arkansas, Delaware, District of Columbia, Florida, Georgia, Kentucky, Louisiana, Maryland, Mississippi, North Carolina, Oklahoma, South Carolina, Tennessee, Texas, Virginia, and West Virginia.) ^§^ Cancer incidence data were compiled from cancer registries that met the data quality criteria for all invasive cancer sites combined, representing approximately 99% of the U.S. population. (Data from Nevada did not meet *U.S. Cancer Statistics* publication criteria for 2010–2014.) Data for Puerto Rico are included in state-specific analyses but not in U.S. census region analyses.

A total of 354,478 new urinary bladder cancer cases (20.5 per 100,000 persons) were reported in the United States during 2010–2014 (Table 10). Incidence rates were approximately four times higher among men (35.8) than among women (8.8). Rates increased with increasing age and peaked among adults aged ≥80 years (340.8 and 73.2 among men and women aged ≥80 years, respectively). Whites had the highest rates (21.8), followed by blacks (11.7), AI/ANs (9.0), and A/PIs (8.5). Rates were higher among non-Hispanics (21.3) than Hispanics (11.2). Among those with known tumor characteristics (97.8%), the majority of cases of urinary bladder cancer were transitional cell carcinomas (94.9%) and were diagnosed at a localized stage (85.6%). Among men during 2010–2014, urinary bladder cancer incidence rates were higher in nonmetropolitan counties (36.2) than in metropolitan counties (35.7). Among women, bladder cancer incidence rates did not differ between nonmetropolitan and metropolitan counties. Among men and women, bladder cancer incidence rates were highest in the Northeast (42.5 and 11.0, respectively) and lowest in the West (32.5 and 7.8, respectively). Connecticut, Delaware, Maine, Massachusetts, New Hampshire, New Jersey, New York, Pennsylvania, and Rhode Island had the highest rates of urinary bladder cancer among men (40.5–48.1) and women (10.5–12.8) (Figures 19 and 20).

**TABLE 10:** Incidence rates* and percentages^†^ of invasive urinary bladder cancer, by demographic and tumor characteristics — United States,^§^ 2010–2014

Demographic characteristic	Total		Male		Female	
	No.	Rate (95% CI)	No.	Rate (95% CI)	No.	Rate (95% CI)
**Total**	**354,478**	**20.5 (20.4–20.6)**	**268,872**	**35.8 (35.7–35.9)**	**85,606**	**8.8 (8.8–8.9)**
**Age group at diagnosis (yrs)**						
<40	3,145	0.4 (0.4–0.4)	2,170	0.6 (0.5–0.6)	975	0.3 (0.2–0.3)
40–49	10,369	4.8 (4.7–4.9)	7,491	6.9 (6.8–7.1)	2,878	2.6 (2.5–2.7)
50–59	42,345	19.2 (19.1–19.4)	31,916	29.7 (29.4–30.1)	10,429	9.3 (9.1–9.5)
60–69	90,204	58.4 (58.0–58.8)	69,828	95.2 (94.5–95.9)	20,376	25.1 (24.7–25.4)
70–79	108,913	125.4 (124.6–126.1)	84,699	217.5 (216.0–218.9)	24,214	50.6 (49.9–51.2)
≥80	99,502	172.4 (171.3–173.5)	72,768	340.8 (338.3–343.2)	26,734	73.2 (72.3–74.1)
**Race**						
White	322,431	21.8 (21.7–21.9)	246,446	38.0 (37.8–38.1)	75,985	9.3 (9.2–9.3)
Black	19,647	11.7 (11.5–11.9)	13,174	19.5 (19.1–19.9)	6,473	6.6 (6.4–6.8)
American Indian/Alaska Native	1,155	9.0 (8.4–9.5)	849	15.3 (14.1–16.4)	306	4.2 (3.7–4.7)
Asian/Pacific Islander	6,084	8.5 (8.3–8.7)	4,558	14.9 (14.5–15.4)	1,526	3.8 (3.6–4.0)
**Ethnicity^¶^**						
Hispanic	15,308	11.2 (11.0–11.3)	11,365	19.5 (19.1–19.9)	3,943	5.0 (4.9–5.2)
Non-Hispanic	339,122	21.3 (21.3–21.4)	257,478	37.2 (37.1–37.4)	81,644	9.2 (9.1–9.2)
**County classification**						
Metropolitan	284,262	20.4 (20.3–20.4)	214,733	35.7 (35.5–35.9)	69,529	8.8 (8.8–8.9)
Nonmetropolitan	53,332	21.0 (20.8–21.1)	41,193	36.2 (35.8–36.6)	12,139	8.8 (8.6–9.0)
**Census region**						
Northeast	81,032	24.3 (24.2–24.5)	60,232	42.5 (42.2–42.9)	20,800	11.0 (10.8–11.1)
Midwest	84,088	21.7 (21.6–21.9)	63,670	37.9 (37.6–38.2)	20,418	9.4 (9.3–9.5)
South	121,179	18.8 (18.7–18.9)	92,425	33.0 (32.8–33.2)	28,754	8.0 (7.9–8.1)
West	68,179	18.7 (18.6–18.9)	52,545	32.5 (32.3–32.8)	15,634	7.8 (7.6–7.9)
Tumor characteristic**	**No.**	**%**	**No.**	**%**	**No.**	**%**
**Total**	**346,522**	**100.0**	**263,415**	**100.0**	**83,107**	**100.0**
Histology						
Transitional cell carcinoma	328,814	94.9	251,579	95.5	77,235	92.9
Squamous cell carcinoma	5,506	1.6	3,170	1.2	2,336	2.8
Adenocarcinoma	3,829	1.1	2,551	1.0	1,278	1.5
Epithelial carcinoma, NOS	6,249	1.8	4,645	1.8	1,604	1.9
All other histologies	2,124	0.6	1,470	0.6	654	0.8
**Stage^††^**						
Localized	296,493	85.6	227,058	86.2	69,435	83.5
Regional	25,399	7.3	18,696	7.1	6,703	8.1
Distant	14,366	4.1	10,047	3.8	4,319	5.2
Unknown	10,264	3.0	7,614	2.9	2,650	3.2

**Abbreviations:** CI = confidence interval; NOS = not otherwise specified.

* New cases diagnosed per 100,000 persons, age adjusted to the 2000 U.S. standard population.

^†^ New cases diagnosed.

^§^ Cancer incidence data were compiled from cancer registries that met the data quality criteria for all invasive cancer sites combined, representing approximately 99% of the U.S. population. (Data from Nevada did not meet *U.S. Cancer Statistics* publication criteria for 2010–2014.)

^¶^ Ethnicity is not mutually exclusive from race.

** Only includes microscopically confirmed cases; excludes cases identified only through death certificate or autopsy report.

^††^ Localized: cancer that is confined to the primary site; regional: cancer that has spread directly beyond the primary site or to regional lymph nodes; distant: cancer that has spread to other organs.

### Cervical Cancer

**FIGURE 21:**
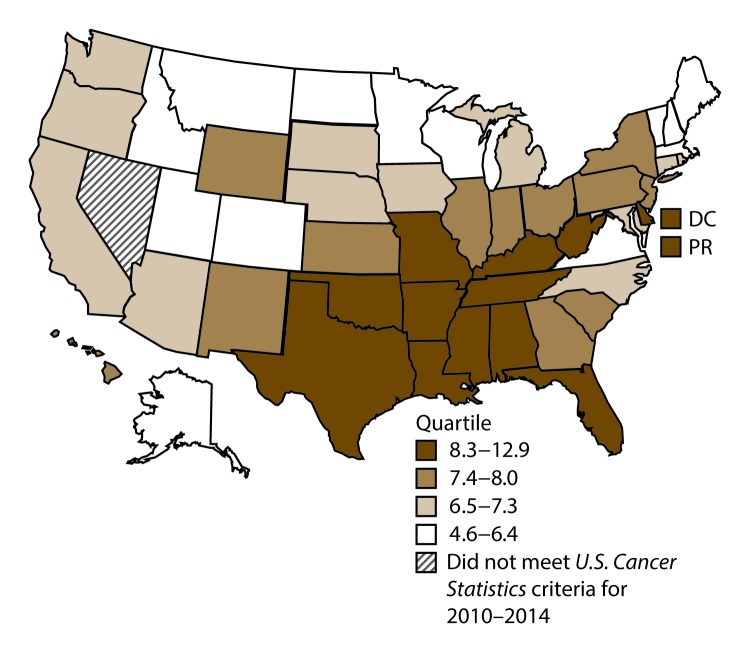
Incidence rates* for cervical cancer, by state/area and U.S. census region^†^ — United States,^§^ 2010–2014 **Abbreviations:** DC = District of Columbia; PR = Puerto Rico. * New cases diagnosed per 100,000 females, age adjusted to the 2000 U.S. standard population. ^†^ *West:* 6.9; *Midwest:* 7.1; *Northeast:* 7.1; *South:* 8.3. (*West:* Alaska, Arizona, California, Colorado, Hawaii, Idaho, Montana, Oregon, New Mexico, Utah, Washington, and Wyoming; *Midwest:* Illinois, Indiana, Iowa, Kansas, Michigan, Minnesota, Missouri, Nebraska, North Dakota, Ohio, South Dakota, and Wisconsin; *Northeast:* Connecticut, Maine, Massachusetts, New Hampshire, New Jersey, New York, Pennsylvania, Rhode Island, and Vermont; *South:* Alabama, Arkansas, Delaware, District of Columbia, Florida, Georgia, Kentucky, Louisiana, Maryland, Mississippi, North Carolina, Oklahoma, South Carolina, Tennessee, Texas, Virginia, and West Virginia.) ^§^ Cancer incidence data were compiled from cancer registries that met the data quality criteria for all invasive cancer sites combined, representing approximately 99% of the U.S. population. (Data from Nevada did not meet *U.S. Cancer Statistics* publication criteria for 2010–2014.) Data for Puerto Rico are included in state-specific analyses but not in U.S. census region analyses.

A total of 61,499 new cervical cancer cases (7.5 per 100,000 women) were reported in the United States during 2010–2014 (Table 11). Women aged 40–49 years had the highest rates of cervical cancer (14.3). Rates were highest among blacks (9.3), followed by whites (7.3), AI/ANs (6.5), and A/PIs (6.1). Rates were higher among Hispanics (9.7) than among non-Hispanics (7.3). Among those with known tumor characteristics (97.1%), the majority of cervical cancer cases were squamous cell carcinomas (66.1%); adenocarcinomas accounted for 27.6%. Most cervical cancer cases were diagnosed at the localized (43.8%) or regional (36.2%) stage. During 2010–2014, cervical cancer rates were higher in nonmetropolitan counties (8.4) than in metropolitan counties (7.4). Cervical cancer cases were highest in the South (8.3) and lowest in the Northeast, Midwest, and West (6.9–7.1). Alabama, Arkansas, DC, Florida, Kentucky, Louisiana, Mississippi, Oklahoma, Puerto Rico, Texas, and West Virginia had the highest rates of cervical cancers (8.6–12.9) (Figure 21).

**TABLE 11:** Incidence rates* and percentages^†^ of invasive cervical cancer, by demographic and tumor characteristics — United States,^§^ 2010–2014

Demographic characteristic	No.	Rate (95% CI)
**Total**	**61,499**	**7.5 (7.5–7.6)**
**Age group at diagnosis (yrs)**		
<40	15,061	3.9 (3.8–3.9)
40–49	15,242	14.3 (14.1–14.6)
50–59	13,551	12.3 (12.1–12.6)
60–69	9,538	11.6 (11.3–11.8)
70–79	5,003	10.4 (10.1–10.7)
≥80	3,104	8.6 (8.3–8.9)
**Race**		
White	47,007	7.3 (7.2–7.4)
Black	9,837	9.3 (9.1–9.5)
American Indian/Alaska Native	609	6.5 (6.0–7.1)
Asian/Pacific Islander	2,900	6.1 (5.9–6.3)
**Ethnicity^¶^**		
Hispanic	10,236	9.7 (9.5–9.9)
Non-Hispanic	51,259	7.3 (7.2–7.3)
**County classification**		
Metropolitan	50,808	7.4 (7.4–7.5)
Nonmetropolitan	8,440	8.4 (8.2–8.6)
**Census region**		
Northeast	10,935	7.1 (6.9–7.2)
Midwest	12,530	7.1 (7.0–7.3)
South	25,587	8.3 (8.2–8.4)
West	12,447	6.9 (6.8–7.0)
Tumor characteristic**	**No.**	**%**
**Total**	**59,726**	**100.0**
**Histology**		
Squamous cell carcinoma	39,480	66.1
Adenocarcinoma	16,456	27.6
Epithelial carcinoma, NOS	2,235	3.7
All other histologies	1,555	2.6
**Stage^††^**		
Localized	26,183	43.8
Regional	21,650	36.2
Distant	8,824	14.8
Unknown	3,069	5.1

**Abbreviations:** CI = confidence interval; NOS = not otherwise specified.

* New cases diagnosed per 100,000 females, age adjusted to the 2000 U.S. standard population.

^†^ New cases diagnosed.

^§^ Cancer incidence data were compiled from cancer registries that met the data quality criteria for all invasive cancer sites combined, representing approximately 99% of the U.S. population. (Data from Nevada did not meet *U.S. Cancer Statistics* publication criteria for 2010–2014.)

^¶^ Ethnicity is not mutually exclusive from race.

** Only includes microscopically confirmed cases; excludes cases identified only through death certificate or autopsy report.

^††^ Localized: cancer that is confined to the primary site; regional: cancer that has spread directly beyond the primary site or to regional lymph nodes; distant: cancer that has spread to other organs.

### Acute Myeloid Leukemia

**FIGURE 22:**
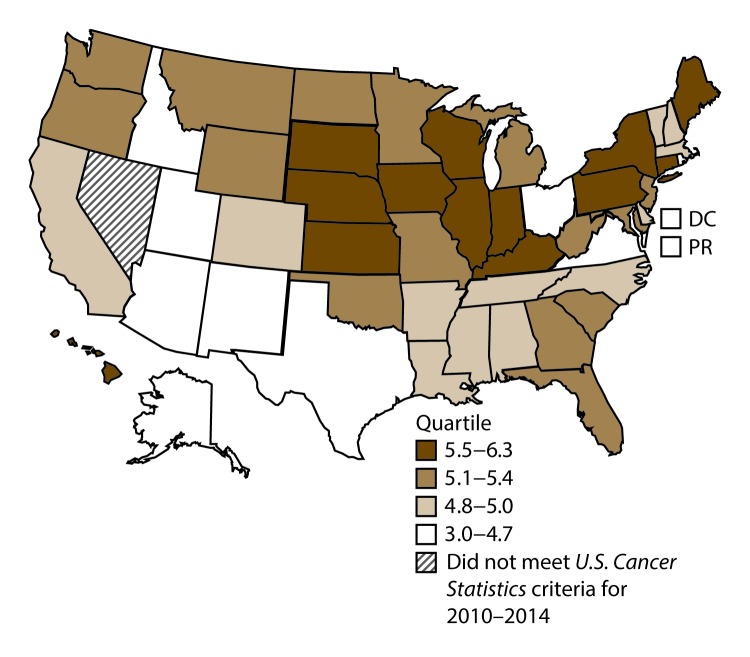
Incidence rates* for male acute myeloid leukemia, by state/area and U.S. census region^†^ — United States,^§^ 2010–2014 **Abbreviations:** DC = District of Columbia; PR = Puerto Rico. * New cases diagnosed per 100,000 males, age adjusted to the 2000 U.S. standard population. ^†^ *West:* 4.9; *Midwest:* 5.5; *Northeast:* 5.5; *South:* 5.0. (*West:* Alaska, Arizona, California, Colorado, Hawaii, Idaho, Montana, Oregon, New Mexico, Utah, Washington, and Wyoming; *Midwest:* Illinois, Indiana, Iowa, Kansas, Michigan, Minnesota, Missouri, Nebraska, North Dakota, Ohio, South Dakota, and Wisconsin; *Northeast:* Connecticut, Maine, Massachusetts, New Hampshire, New Jersey, New York, Pennsylvania, Rhode Island, and Vermont; *South:* Alabama, Arkansas, Delaware, District of Columbia, Florida, Georgia, Kentucky, Louisiana, Maryland, Mississippi, North Carolina, Oklahoma, South Carolina, Tennessee, Texas, Virginia, and West Virginia.) ^§^ Cancer incidence data were compiled from cancer registries that met the data quality criteria for all invasive cancer sites combined, representing approximately 99% of the U.S. population. (Data from Nevada did not meet *U.S. Cancer Statistics* publication criteria for 2010–2014.) Data for Puerto Rico are included in state-specific analyses but not in U.S. census region analyses.

**FIGURE 23:**
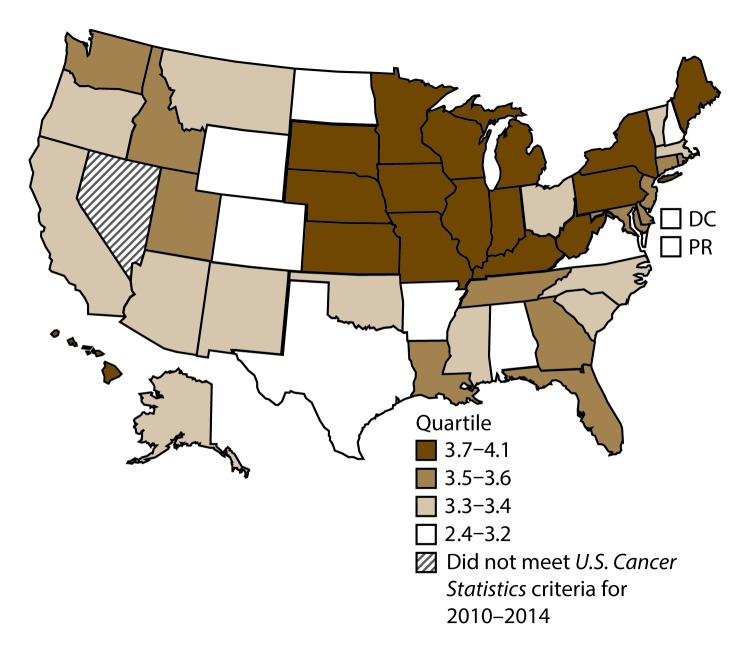
Incidence rates* for female acute myeloid leukemia, by state/area and U.S. census region^†^ — United States,^§^ 2010–2014 **Abbreviations:** DC = District of Columbia; PR = Puerto Rico. * New cases diagnosed per 100,000 females, age adjusted to the 2000 U.S. standard population. ^†^ *West:* 3.4; *Midwest:* 3.7; *Northeast:* 3.7; *South:* 3.4. (*West:* Alaska, Arizona, California, Colorado, Hawaii, Idaho, Montana, Oregon, New Mexico, Utah, Washington, and Wyoming; *Midwest:* Illinois, Indiana, Iowa, Kansas, Michigan, Minnesota, Missouri, Nebraska, North Dakota, Ohio, South Dakota, and Wisconsin; *Northeast:* Connecticut, Maine, Massachusetts, New Hampshire, New Jersey, New York, Pennsylvania, Rhode Island, and Vermont; *South:* Alabama, Arkansas, Delaware, District of Columbia, Florida, Georgia, Kentucky, Louisiana, Maryland, Mississippi, North Carolina, Oklahoma, South Carolina, Tennessee, Texas, Virginia, and West Virginia.) ^§^ Cancer incidence data were compiled from cancer registries that met the data quality criteria for all invasive cancer sites combined, representing approximately 99% of the U.S. population. (Data from Nevada did not meet *U.S. Cancer Statistics* publication criteria for 2010–2014.) Data for Puerto Rico are included in state-specific analyses but not in U.S. census region analyses.

A total of 70,960 new AML cases (4.2 per 100,000 persons) were reported in the United States during 2010–2014 (Table 12). Incidence rates were higher among men than among women (5.2 and 3.5, respectively). Rates increased with increasing age and peaked among adults aged ≥80 years (26.3). Whites had the highest rates (4.3), followed by blacks (3.5), A/PIs (3.4), and AI/ANs (2.7). Non-Hispanics had higher rates than Hispanics (4.3 and 3.6, respectively). During 2010–2014, AML incidence rates were similar between metropolitan and nonmetropolitan counties (4.2). Among men and women, AML incidence rates were highest in the Northeast and Midwest (5.5 among men and 3.7 among women) and lowest in the South and West (4.9–5.0 among men and 3.4 among women). Hawaii, Illinois, Indiana, Iowa, Kentucky, Maine, Pennsylvania, South Dakota, and Wisconsin had some of the highest rates among men (5.6–6.3) and women (3.7–4.1) (Figures 22 and 23).

**TABLE 12:** Incidence rates* and percentages^†^ of acute myeloid leukemia, by demographic and tumor characteristics — United States,^§^ 2010–2014

Demographic characteristic	Total		Male		Female	
	No.	Rate (95% CI)	No.	Rate (95% CI)	No.	Rate (95% CI)
**Total**	**70,960**	**4.2 (4.2–4.2)**	**39,024**	**5.2 (5.1–5.2)**	**31,936**	**3.5 (3.5–3.6)**
**Age group at diagnosis (yrs)**						
<40	8,576	1.0 (1.0–1.1)	4,254	1.0 (1.0–1.1)	4,322	1.1 (1.0–1.1)
40–49	4,967	2.3 (2.3–2.4)	2,512	2.4 (2.3–2.5)	2,455	2.3 (2.2–2.4)
50–59	9,408	4.3 (4.2–4.4)	5,099	4.8 (4.7–4.9)	4,309	3.9 (3.8–4.0)
60–69	15,497	10.0 (9.8–10.2)	9,068	12.3 (12.1–12.6)	6,429	7.9 (7.7–8.1)
70–79	17,344	20.0 (19.7–20.3)	10,119	26.0 (25.5–26.5)	7,225	15.1 (14.7–15.4)
≥80	15,168	26.3 (25.9–26.8)	7,972	37.3 (36.5–38.1)	7,196	19.9 (19.4–20.3)
**Race**						
White	60,923	4.3 (4.3–4.3)	33,980	5.3 (5.2–5.3)	26,943	3.6 (3.5–3.6)
Black	6,368	3.5 (3.4–3.6)	3,157	4.1 (4.0–4.3)	3,211	3.1 (3.0–3.2)
American Indian/Alaska Native	432	2.7 (2.5–3.0)	214	3.1 (2.6–3.6)	218	2.5 (2.2–2.9)
Asian/Pacific Islander	2,645	3.4 (3.2–3.5)	1,361	4.0 (3.7–4.2)	1,284	2.9 (2.8–3.1)
**Ethnicity^¶^**						
Hispanic	6,362	3.6 (3.5–3.7)	3,351	4.2 (4.0–4.4)	3,011	3.2 (3.0–3.3)
Non-Hispanic	64,594	4.3 (4.2–4.3)	35,671	5.2 (5.2–5.3)	28,923	3.5 (3.5–3.6)
**County classification**						
Metropolitan	57,611	4.2 (4.2–4.2)	31,583	5.2 (5.1–5.2)	26,028	3.5 (3.5–3.5)
Nonmetropolitan	10,050	4.2 (4.1–4.3)	5,611	5.1 (4.9–5.2)	4,439	3.6 (3.5–3.7)
**Census region**						
Northeast	14,211	4.5 (4.4–4.5)	7,763	5.5 (5.4–5.6)	6,448	3.7 (3.6–3.8)
Midwest	16,751	4.5 (4.4–4.5)	9,143	5.5 (5.3–5.6)	7,608	3.7 (3.7–3.8)
South	25,375	4.0 (4.0–4.1)	14,003	5.0 (4.9–5.0)	11,372	3.4 (3.3–3.4)
West	14,623	4.0 (4.0–4.1)	8,115	4.9 (4.8–5.0)	6,508	3.4 (3.3–3.5)

**Abbreviation:** CI = confidence interval.

* New cases diagnosed per 100,000 persons, age adjusted to the 2000 U.S. standard population.

^†^ New cases diagnosed.

^§^ Cancer incidence data were compiled from cancer registries that met the data quality criteria for all invasive cancer sites combined, representing approximately 99% of the U.S. population. (Data from Nevada did not meet *U.S. Cancer Statistics* publication criteria for 2010–2014.)

^¶^ Ethnicity is not mutually exclusive from race.

### Estimated Annual Percentage Change in Incidence Rates of Tobacco-Associated Cancers

During 2010–2014, APCs in incidence rates of tobacco-associated cancers varied by site, sex, and men and women combined (Table 13) (Supplementary Table 1, https://stacks.cdc.gov/view/cdc/59431; Supplementary Table 2, https://stacks.cdc.gov/view/cdc/59432). Significant decreases in cancers of the lung (2.17% per year), larynx (3.00%), esophagus (0.86%), stomach (1.01%), urinary bladder (1.28%), and colon and rectum (2.07%) occurred among men and women combined (Table 13). Although esophageal and stomach cancer rates decreased overall, rates remained stable among women. The decrease in lung cancer rates was significantly greater among men (2.88%) than among women (1.47%). Significant increases in liver (1.98% per year) and kidney (0.54%) cancer and AML (0.34%) occurred in men and women combined. Rates for OCP, pancreatic, and cervical cancers were stable during 2010–2014. 

**TABLE 13:** Annual percentage change* in incidence rates^†^ of tobacco-associated cancers, by cancer site and sex — United States,^§^ 2010–2014^¶^

Cancer site	Total		Male		Female	
	APC	p value**	APC	p value	APC	p value
**All tobacco-related cancer sites**	**-1.20**	**<0.001**	**-1.40**	**0.001**	**-1.10**	**<0.001**
Oral cavity and pharynx	0.63	0.09	0.79	0.18	0.06	0.81
Esophagus	-0.86	0.02	-1.12	0.006	-0.54	0.24
Stomach	-1.01	<0.001	-1.25	0.02	-0.92	0.08
Colon and rectum	-2.07	0.002	-2.19	0.001	-2.04	0.005
Liver	1.98	0.02	1.72	0.04	2.43	0.02
Pancreas	0.53	0.08	0.70	0.05	0.36	0.10
Larynx	-3.00	0.004	-3.18	0.008	-2.81	0.004
Trachea, lung, and bronchus	-2.17	<0.001	-2.88	<0.001	-1.47	0.007
Cervix uteri	—	—	—	—	-0.87	0.15
Kidney and renal pelvis	0.54	0.01	0.69	0.004	0.07	0.73
Urinary bladder	-1.28	0.02	-1.51	0.02	-1.29	0.04
Acute myeloid leukemia	0.34	0.03	0.36	0.22	0.17	0.59

**Abbreviations:** APC = annual percentage change.

* Calculated by least squares regression.

^†^ New cases diagnosed per 100,000 persons, age adjusted to the 2000 U.S. standard population.

^§^ Cancer incidence data were compiled from cancer registries that met the data quality criteria for all invasive cancer sites combined, representing approximately 99% of the U.S. population. (Data from Nevada did not meet *U.S. Cancer Statistics* publication criteria for 2010–2014.)

^¶^ See also Supplementary Table 1 (https://stacks.cdc.gov/view/cdc/59431) and Supplementary Table 2 (https://stacks.cdc.gov/view/cdc/59432).

** p<0.05 was considered statistically significant.

## Discussion

### Interpretation of Tobacco-Associated Cancer Incidence

Approximately 3.3 million cases of tobacco-associated cancer were reported in the United States during 2010–2014 (approximately 667,000 each year), with lung cancer accounting for about a third of these diagnoses. The incidence of tobacco-associated cancers was higher among men than women. Black, white, and non-Hispanic populations had consistently higher incidence rates than other racial/ethnic populations, with a few exceptions. The majority of tobacco-associated cancers occurred among persons aged ≥70 years. Except for AML and cancers of the stomach, liver, and pancreas, tobacco-associated cancer rates were higher among nonmetropolitan counties than in metropolitan counties. The high rates among men, whites, blacks, non-Hispanics, older adults, and nonmetropolitan county residents reflect overall demographic patterns of cancer incidence in the United States (*39*) and patterns of tobacco use. In 2016, an estimated 15.5% of adults in the United States (37.8 million persons) smoked cigarettes, with smoking being more common among men (17.5%) than among women (13.5%) (*29*). The prevalence of cigarette smoking in 2016 varied among racial/ethnic groups; the highest prevalence was among AI/ANs (31.8%), followed by persons of multiple races (25.2%), whites (16.6%), blacks (16.5%), Hispanics (10.7%), and A/PIs (9.0%) (*29*). Smoking prevalence among AI/ANs varies by region of the country, and cancer rates tend to parallel these differences (*47*). Blacks and whites had the highest tobacco-associated cancer incidence rates in this report and the second- and third-highest smoking prevalence, respectively, among racial/ethnic populations.

Substantial variation in tobacco use prevalence exists by state among adults in the United States (*48*). During 2014–2015, nine of the 10 states with the highest prevalence of current use of any tobacco product were in either the South (West Virginia [26.9%], Kentucky [26.2%], Arkansas [24.0%], Oklahoma [23.8%], Alabama [23.1%], and Tennessee [22.7%]) or in the Midwest (Ohio [23.8%], South Dakota [23.0%], and North Dakota [22.6%]) (*47*). States with the lowest prevalence of current use of any tobacco product were in the West (California [10.2%] and Utah [10.9%]) (*48*). In this report, lung, laryngeal, OCP, kidney, and cervical cancer incidence rates were highest in the South and the Midwest. Cancer incidence rates were consistently lowest in the West for all the cancers in this report, with the exception of kidney and stomach cancers.

Cancers of the lung and bronchus, larynx, and OCP have the greatest average relative risks (*1*,*22*,*49*) associated with tobacco use. Lung cancer is the second most commonly diagnosed cancer among men (after prostate cancer) and women (after breast cancer) and is the leading cause of cancer death both among men and women in the United States (*39*). The average tobacco-associated lung cancer relative risk ranges from 15.0 to 30.0 (*1*,*22*,*49*). Tobacco use causes all histologic types of lung cancer (*1*,*50*). Similar to previous studies, this study demonstrates that the percentage of squamous cell carcinoma of the lung is higher among men than among women, whereas the percentage of adenocarcinoma of the lung is higher among women than among men (*19*,*51*). In the United States, the decrease in squamous cell carcinoma followed the trend of decreasing smoking prevalence (*1*). Adenocarcinoma rates in the United States increased in the 1980s and 1990s before decreasing through 2004 and reportedly increasing again during 2006–2010; however, much of this latter increase reflects improved classification of tumors previously designated as “other non–small cell lung cancer” (*1*,*52*). The proportion of lung cancers classified as adenocarcinoma has increased over time and adenocarcinoma is now the most common type of lung cancer both among men and women smokers (*1*,*27*).

In 2013, the U.S. Preventive Services Task Force recommended annual screening with low-dose computed tomography (LDCT) for persons at high risk for lung cancer (i.e., adults aged 55–80 years who have a history of smoking 30 packs per year and currently smoke or have quit within the past 15 years) (*53*). However, the percentage of persons screened has been low, with 3.3% of eligible smokers reporting LDCT screening in the past 12 months in 2010 and 3.9% in 2015 (*54*). The reasons for underuse of screening might include smokers’ lack of awareness about this test and limited health care access, as well as physicians’ lack of knowledge about screening recommendations and concerns about false-positive results (*55*). Some studies have reported that eligible patients who benefit most from screening are those with chronic obstructive pulmonary disease (COPD) (*56*,*57*). Compared with patients with normal baseline spirometry who receive annual screening with LDCT, patients with COPD have a greater incidence of lung cancer and are more likely to be detected with early stage disease (*56*,*57*). Additional research is needed to determine whether current screening recommendations can be modified to include COPD status.

Laryngeal cancer is relatively rare in the United States (*39*). The average tobacco-associated relative risk for laryngeal cancer ranges from 10.0 to 17.0 (*1*,*22*,*49*). Tobacco use is the single most important risk factor for developing laryngeal cancer (*1*,*2*,*7*). The decrease in laryngeal cancer incidence rates observed in this study among men and women is consistent with decreasing incidence rates reported outside the United States (*58*). The decrease might be partially explained by changes in tobacco use (*1*). Alcohol use also is a risk factor for laryngeal cancer, and when used together, alcohol and tobacco have a synergistic effect on laryngeal cancer development (*26*,*59*). However, the prevalence of alcohol consumption among U.S. adults has not decreased substantially in recent years (*60*). Decreased dietary fiber intake also might be associated with risk for laryngeal cancer, particularly among women (*61*–*63*).

OCP cancer is the ninth most common cancer in the United States among men but is less common among women (*39*). This type of cancer includes tumors with origins in several anatomic organs of the head and neck. The average relative risk for tobacco-associated OCP cancer is 4.0–5.0 (*1*,*22*,*49*). Use of all tobacco products, including cigarettes, cigars, pipes, and smokeless tobacco, are linked to head and neck cancer (*2*,*7*,*28*). Strong evidence implicates tobacco as a carcinogenic factor in squamous cell cancers of the head and neck (*1*,*28*). The predominant histologic tumor type of OCP cancer observed in this study was squamous cell carcinoma. Previous studies have demonstrated that cessation of tobacco use leads to a decrease in the risk for OCP cancer (*64*). Alcohol use is a risk factor for the development of OCP cancer, independent of the effects of tobacco use (*65*–*67*). The use of both alcohol and tobacco have a synergistic effect on the development of OCP cancers (*26*), accounting for as many as 64% of oral cavity and 72% of pharyngeal cancers (*59*,*65*,*68*). Infection with human papillomavirus (HPV), particularly HPV16, increases the risk for cancer at certain OCP sites, including the oropharynx, base of the tongue, and tonsils (*69*,*70*).

Cancers of the pancreas, urinary bladder, and colon and rectum have similar relative risks associated with tobacco use (pancreas, 2.0–4.0; urinary bladder, 3.0; colon and rectum, 1.9–2.5) (*1*,*22*,*49*,*71*). Pancreatic cancer is among the 10 most common cancers diagnosed in the United States and is the fifth leading cause of cancer death among men and women in the United States (*39*). Although recent increases in pancreatic cancer incidence might be due to increases in the prevalence of excess body weight (*72*), previous decreases were attributed to decreases in smoking prevalence (*1*). One study indicated that cigarette smoke increased tumor growth and metastases in pancreatic cancer cells (*73*). Human and animal studies have found that functional nicotinic receptors are present on pancreatic islet and beta cells, and nicotine can reduce the release of insulin through neuronal nicotinic acetylcholine receptors on islet cells (*74*). The collection of multiple studies in animals and humans strongly supports the hypothesis that cigarette smoking and exposure to nicotine can adversely affect insulin action and the function of pancreatic cells, both of which play fundamental roles in the pathogenesis of diabetes (*75*). Being overweight or having obesity, having a personal history of diabetes or chronic pancreatitis, and several genetic syndromes also are associated with increased risk for pancreatic cancer (*76*,*77*). Findings from a recent meta-analysis show that increased fruit and vegetable intake is associated with decreased risk for pancreatic cancer (*78*).

Urinary bladder cancer is the fourth most commonly diagnosed cancer and the seventh leading cause of cancer death among men in the United States (*39*). Among women, urinary bladder cancer is much less common. The average tobacco-associated bladder cancer relative risk is 4.0 (*22*,*49*). The decrease in bladder cancer incidence rates reported in this study among men and women is consistent with findings from other studies (*19*,*79*). The incidence of urinary bladder cancer has decreased among men and women in the United States since 1999 (*79*). The strongest risk factor for bladder cancer in the United States is cigarette smoking. Approximately half of all bladder cancers are caused by cigarette smoking (*80*–*82*). Quitting smoking decreases the risk for bladder cancer by approximately 40% within 1–4 years (*80*,*81*,*83*). Pipe and cigar use (among noncigarette smokers) also is associated with increased risk for bladder cancer among men (*81*).

Colorectal cancer (i.e., cancer of the colon and rectum) is the second most common cancer diagnosed and the second leading cause of cancer death among men and women combined in the United States (*39*). Colorectal cancer is one of two additional cancers (including liver cancer) that were causally linked to tobacco use in the 2014 Surgeon General’s report (*1*). The incidence of colorectal cancer has decreased steadily since 2001; most recently, incidence decreased an average of 3% annually during 2005–2014 (*84*). This decrease might be due to increased colorectal cancer screening through the detection of precancerous polyps, which can then be removed before becoming cancerous, thus decreasing cancer incidence (*72*,*85*). Risk for colorectal cancer has been reported to increase with increased daily cigarette consumption and duration of smoking (*86*,*87*). Long-term cigarette smoking is associated with increased incidence of colorectal cancer and risk for death from colorectal cancer in men and women (*1*,*27*). In a study examining metabolites of tobacco smoking and colorectal cancer risk, persons with detectable levels of serum hydroxycotinine were reported to have a greater risk than those with undetectable levels (*88*). Additional research using biomarkers for smoking behaviors might help refine estimates of the association between cigarette smoking and colorectal cancer risk. An increased risk for colorectal cancer is associated with excessive alcohol use (*89*), being overweight or having obesity (*90*), consumption of processed or red meat (*91*), and certain genetic factors. Physical activity and fiber consumption might reduce the risk for colorectal cancer (*92*).

AML, esophageal, kidney, stomach, liver, and cervical cancers have similar relative risks associated with tobacco use (1.5–2.5) (*1*,*22*,*49*). Esophageal cancer is not as commonly diagnosed in the United States as other cancers included in this report; however, esophageal cancer is one of the top 10 leading causes of cancer death among most racial/ethnic male populations (*39*). Squamous cell carcinoma of the esophagus was the more prevalent histologic subtype in the United States until the mid-1990s, when the incidence of squamous cell carcinoma decreased while the incidence of adenocarcinoma increased, resulting in adenocarcinoma becoming the most common histologic subtype (*93*–*95*). Current smokers have a three to five times higher risk for developing squamous cell carcinoma compared with never smokers, and former smokers have a significantly lower risk for developing this subtype compared with current smokers (*96*,*97*). Increased time since quitting smoking is associated with decreased risk for squamous cell carcinomas (*96*,*97*). The incidence of squamous cell carcinoma of the esophagus has decreased sharply in North America since the early 1970s, which is likely due to lower rates of smoking (*96*,*98*,*99*). In contrast, during this same period, the incidence of adenocarcinoma of the esophagus has increased (*95*). Both current and former smokers have approximately twice the risk for developing this subtype as never smokers (*96*,*97*). Esophageal adenocarcinoma also is associated with obesity and alcohol use (*96*). The prevalence of obesity among U.S. adults aged ≥20 years increased from 1999–2000 through 2015–2016 (*100*). Obesity has been most strongly associated with early onset (among persons aged <50 years) esophageal adenocarcinoma (*101*). In 2016, 55.0% of adults in the United States reported drinking alcohol in the past month, a slight increase from 1997 (53.5%) (*102*).

Kidney cancer is among the 10 most common cancers diagnosed both among men and women in the United States (*39*). Smoking is an important risk factor for kidney cancers (*103*). Cohort studies have reported positive associations between smoking and kidney cancer incidence (*104*,*105*) and death (*106*,*107*). In addition to smoking, excess body weight, and hypertension are risk factors for kidney cancer (*104*,*108*,*109*). Both for men and women, incidence rates of kidney cancer during 2010–2014 were higher compared with previously published estimates for 1999–2004 (*19*). Certain studies suggest that these increases might have resulted from increased detection of kidney cancer through recent increased use of ultrasound and computed tomography for non–cancer-related conditions (*110*–*112*) or from increases in the prevalence of overweight and obesity (*42*).

Stomach cancer is common among certain racial/ethnic populations in the United States (*39*). Among current smokers, risk for stomach cancer increases with the number of cigarettes smoked per day and duration of smoking (*113*). The risk for stomach cancer decreases as time since stopping smoking increases (*113*). Tobacco use is related to both cardia and noncardia stomach cancer (*96*). Former, light, and moderate cigarette smokers have slightly higher risks for developing gastric cardia cancer than noncardia cancer (*113*). The incidence rate of stomach cancer in this report was higher among A/PIs, non-Hispanic blacks, and Hispanics compared with non-Hispanic whites. The finding in this report that stomach cancer incidence has been decreasing in recent years is consistent with a decrease that has been occurring since the middle of the 20th century in high-income countries in North America and Western Europe and also more recently in areas with historically high rates of stomach cancer (e.g., Asia and Latin America) (*114*). Trends in decreasing stomach cancer rates might be attributable to decreased prevalence of *Helicobacter pylori* infection resulting from improved sanitation and use of antibiotics, increased availability of fresh produce, and lower consumption of salt-preserved and smoked food (*92*,*115*). In addition, decreases in smoking (in countries with high rates of tobacco use) might have contributed to the decreasing trends of stomach cancer incidence (*116*). *H. pylori* is a major risk factor for noncardia gastric cancer; therefore, the decrease in stomach cancer incidence largely reflects decreasing occurrence at this anatomic subsite (*114*,*117*). In contrast to noncardia gastric cancer, rates of cardia gastric cancer are increasing in the United States and other high-income countries (*114*). This increase might be associated with increases in obesity or improvements in classification of stomach tumors (*118*).

Liver cancer is among the 10 most common cancers diagnosed among nonwhite men in the United States and the fifth leading cause of cancer death among all men in the United States (*39*). Although liver cancer occurs more frequently in less developed regions of the world (*1*,*119*), this cancer type is still a substantial health concern in the United States. Liver cancer is one of two additional cancers (including colorectal) that was causally linked to tobacco use in the 2014 Surgeon General’s report (*1*). Approximately 30,000 new cases of liver cancer are diagnosed every year in the United States, with approximately 20,000 associated deaths (*1*,*39*). In addition to cigarette smoking, metabolic disorders (including obesity), infection with hepatitis B virus or hepatitis C virus, and alcohol use are risk factors for developing liver cancer (*120*). Long-term exposure to carcinogens in smoke might cause cellular damage in the liver and contribute to the development of cancer (*1*). Smoking has also been recognized as a risk factor for primary biliary cirrhosis, a condition that can progress to hepatocellular carcinoma, or primary liver cancer (*121*–*123*), and smoking cessation has benefitted patients with chronic liver diseases (*122*). A 2009 meta-analysis (*124*) showed an estimated 50% increased risk for liver cancer among current smokers compared with never smokers (i.e., adults who have never smoked or who have smoked <100 cigarettes in their lifetime).

Although the overall incidence of cervical cancer is low in the United States, black and Hispanic women continue to have a higher incidence than white women (*39*). Smoking has been consistently associated with an increased risk for cervical cancer, and risk increases with increased daily cigarette smoking and duration of smoking (*125*–*127*). Although tobacco use is associated with cervical cancer, the primary risk factor is HPV infection, which is thought to be responsible for approximately 90% of cervical cancers (*128*). In this report, HPV infection likely contributed to the lower incidence rate among women aged 40–49 years, as well as the high rate among black and Hispanic women (*128*,*129*). The combination of smoking and HPV infection might increase the risk, such that women who are HPV-positive and smoke have a twofold increased risk for cervical cancer compared with women who are HPV-positive and have never smoked (*126*,*127*). Women who have quit smoking cigarettes for long periods have a decreased risk for cervical cancer compared with current smokers, even after adjusting for the presence of an HPV infection (*126*). Cervical cancer is largely preventable with the HPV vaccine, current recommendations are to routinely vaccinate girls and boys aged 11–12 years; women up to age 26 years and men up age 21 years also might be vaccinated if they were not previously (*130*). Routine screening remains an important part of cervical cancer prevention (*131*,*132*). Two tests can be used for cervical cancer screening: a Papanicolaou (Pap) test and an HPV test; one or both of these tests is recommended, depending on the age of the woman (*132*). Special outreach efforts to increase cervical cancer screening among black and Hispanic women are underway (*131*). Targeted education about the dangers of tobacco use in these populations might also be warranted.

AML is a relatively rare cancer in the United States (*39*). Cigarette smoking is an established risk factor for AML in adults (*133*,*134*). One of the carcinogens contained in cigarette smoke is benzene, which is strongly associated with increased risk for AML (*3*). Current smokers have a 40% increased risk for developing AML compared with nonsmokers (*134*). AML risk also increases with higher intensity (i.e., number of cigarettes smoked daily) and longer duration (i.e., number of years) of smoking (*134*). Smoking cessation might decrease AML risk (*135*). In this report, AML incidence rates were highest among white men and women, and the Midwest and Northeast had the highest incidence rates among men. A previous report noted U.S. state incidence rates of AML were inconsistent with smoking patterns (*19*). In this report, many states with the highest observed AML incidence rates among men (i.e., Connecticut, Hawaii, Illinois, Iowa, Maine, New York, Pennsylvania, and Wisconsin) also were not those with the highest rates of smoking in the United States (*48*). The etiology for most cases of AML is unclear. Other environmental exposures and genetic risk factors might play a role (*136*).

### Public Health Implications

Implementing tobacco control policies and programs as recommended by *Ending the Tobacco Epidemic: A Tobacco Control Strategic Action Plan* by the U.S. Department of Health and Human Service (*137*) and *Ending the Tobacco Problem: A Blueprint for the Nation* by the National Academy of Medicine (NAM, formerly the Institute of Medicine) (*138*) would accelerate the decrease of tobacco use among youths and adults and accelerate progress toward achieving the *Healthy People 2020* objectives to reduce cigarette smoking (*1*). The NAM report presented a blueprint for action to reduce tobacco use to a level that would eliminate smoking as a public health problem. The two-pronged strategy for achieving this goal included 1) strengthening and fully implementing traditional tobacco control measures and 2) changing the legal framework to permit policy innovations. Sustained implementation of comprehensive state tobacco control programs can accelerate progress toward reducing adult smoking prevalence (*32*,*139*).

CDC recommends that each state establish and sustain a comprehensive tobacco control program that contains the following overarching components: state and community interventions; health communication interventions that reach large audiences (i.e., mass-reach interventions); cessation interventions; surveillance and evaluation; and infrastructure, administration, and management (*139*). Evidence-based statewide tobacco control programs that are comprehensive, sustained, and accountable have decreased smoking rates and tobacco-associated diseases and deaths (*139*). A comprehensive statewide tobacco control program is a coordinated effort to establish smoke-free policies and social norms, promote and assist with cessation of tobacco, and prevent initiation of tobacco use. CDC’s *Best Practices for Comprehensive Tobacco Control Programs*—*2014* is an evidence-based guide to help states plan and establish comprehensive tobacco control programs (*139*). The report describes an integrated programmatic structure for implementing effective interventions and estimates the recommended level of state investment to reach these goals and to reduce tobacco use in each state. Additional support for tobacco prevention and control is available from CDC’s National Comprehensive Cancer Control Program (NCCCP), which in 1998 began providing financial support and technical assistance to states, tribes, and territories to create, implement, and evaluate cancer control plans (*140*). A 2013 analysis of 69 NCCCP cancer plans showed that every plan contained at least one CDC-recommended, evidence-based tobacco control strategy (*141*). Strategies incorporated were consistent with CDC’s *Best Practices for Comprehensive Tobacco Control Programs* or *The* *Guide to Community Preventive Services* (*141*). CDC also funds the Consortium of National Networks to Impact Populations Experiencing Tobacco-Related and Cancer Health Disparities and the National Tobacco Control Program, which both promote tobacco use prevention and cancer prevention among persons at highest risk for tobacco use (*140*,*142*).

Increased state investments in tobacco control programs, compared with the overall U.S. investments, are more positively correlated with decreases in cigarette sales and the prevalence of smoking (*143*–*145*). For example, during 2001–2010, decreases in smoking prevalence among adults and youths in New York (as reported by the New York State Tobacco Control Program) were higher than overall U.S. decreases in smoking prevalence (*146*). As a result, smoking-attributable personal health care expenditures in New York in 2010 were $4.1 billion less than they would have been had the prevalence of smoking remained at 2001 levels (*146*). In addition to benefits of larger investments in tobacco control programs on smoking rates, research also shows that the longer states invest in such programs, the greater and more rapid the impact (*138*,*139*). For example, in California, which has the nation’s first and longest-running comprehensive tobacco control program, the prevalence of smoking among adults decreased from 22.7% in 1988 to 10.5% in 2015 (*147*). Correspondingly, lung cancer incidence has decreased nearly four times faster in California than in the rest of the United States (*148*). Other states such as Colorado (*149*), Florida (*150*), Massachusetts (*151*), and Minnesota (*152*) also have decreased smoking among adults and youths through various approaches. Data from continued monitoring of cancer incidence rates and death rates at the local, state, and national levels can be used to evaluate the impact of these and other public health successes in reducing the effects of tobacco use.

Preventive services recommended by the U.S. Preventive Services Task Force for decreasing tobacco-associated cancers include tobacco cessation counseling and treatment as well as screening for cervical, colorectal, and lung cancers to help detect the diseases at an early, often treatable, stage (*153*). The Advisory Committee for Immunization Practices recommends hepatitis B virus and HPV vaccinations to prevent infection with these viruses that are also known to cause tobacco-associated cancers (liver, cervical, and OCP). NCCCP funds states, DC, tribes, and territories to work through state and local level cancer coalitions to ensure access to these early detection and treatment services, implement evidence-based programs to prevent cancer, and support cancer survivorship activities. Federal initiatives also can help reduce tobacco use and tobacco-associated cancers. For example, a 1997 executive order established a smoke-free environment for federal employees and members of the public visiting or using federal facilities by prohibiting smoking of tobacco products in all interior spaces owned, rented, or leased by the executive branch of the federal government (*154*).

Continued research is important to identify additional types of cancer that might be associated with tobacco use as well as to enhance understanding of how tobacco exposure affects the cellular processes responsible for promotion and progression of the cancers examined in this report. In 2014, the Surgeon General’s report reviewed the evidence on smoking and cancers of prostate and breast; however, evidence to establish causality was inadequate or insufficient (*1*). Among men in the United States, prostate cancer is the most commonly diagnosed cancer and the second leading cause of cancer death (*39*). Evidence suggests that smoking, especially current or recent smoking, is a risk factor for death from prostate cancer but not for incidence, indicating that continued smoking might lead to higher risk for disease progression (*1*). Evidence of a positive association between smoking and prostate cancer deaths but no association with incidence is consistent across prospective cohort studies conducted in various settings and decades (*1*). Prostate cancer mortality is higher among smokers than nonsmokers (*1*). However, the mortality rates in former smokers who quit years in the past suggests that quitting smoking might reduce prostate cancer mortality (*1*). The Surgeon General’s report recommended future research needs to refine the temporal relation between smoking, cessation, and prostate cancer diagnoses and related mortality (*1*).

Breast cancer is the most frequently diagnosed type of cancer (other than nonmelanoma skin cancers) and the second leading cause of cancer death among women in the United States (*39*). The Surgeon General concluded that the evidence suggests but is not sufficient to infer a causal relation between active smoking or secondhand tobacco smoke exposure and breast cancer (*1*). Evidence (including several large cohort studies) suggests a person with a history of ever smoking has a 10% increased likelihood of receiving a breast cancer diagnosis, an association that although significant, is weak (*1*). The International Agency for Research on Cancer similarly concluded that the overall association between smoking and breast cancer is weaker than the association between smoking and other cancers and that the dose-response relations (i.e., pack years, cigarettes smoked per day, and age at initiation) are correspondingly small (*27*). As stated in the 2014 Surgeon General’s report, because breast cancer is the most common cancer type among women, decreases in smoking might have a substantial impact on the overall incidence of breast cancer (*1*).

### Future Directions

This report examines the incidence of tobacco-associated cancers by demographic and tumor characteristics and by state and U.S. census region. Examination of county-level patterns within states might be helpful in determining areas with disproportionately high cancer incidence or death rates. Using tools such as State Cancer Profiles (https://statecancerprofiles.cancer.gov) could help identify prevalence of risk factors and use of screening tests that might affect these rates. Additional research and data evaluation are needed to determine whether a causal relation exists between smoking and cancers of the prostate and breast.

### Limitations

The findings in this report are subject to at least four limitations. First, because information about tobacco use and other risk factors for cancer is not available in all cancer registry data, these results should not be used to describe cancer rates among smokers. Rather, the incidence rates are limited to cancers that are known to be associated with tobacco use. Second, cancer has many different risk factors and combinations of factors, including tobacco use; therefore, the number of cases and trends in tobacco-associated cancers also might be affected by changes in these other risk factors or cancer screening trends. Third, although this report includes estimates of tobacco-associated cancers, it does not estimate what proportion of these cancers are attributable to tobacco use. Although the 2014 Surgeon General’s report was used to define tobacco-associated cancers, not all incident cancers for each cancer site are necessarily caused by smoking; likewise, using this definition might underestimate the true incidence because evidence that tobacco use might cause additional cancers is still accumulating (*1*). Finally, findings related to race/ethnicity might have been affected if race/ethnicity information was incorrectly collected or recorded in the medical record; ongoing procedures are used to ensure that this information is as accurate as possible (*155*). For example, to minimize misclassification of AI/ANs, some previous studies have restricted analyses to Contract Health Service Delivery Area (CHSDA) counties and have found that rates for AI/ANs in CHSDA counties are generally higher than for AI/ANs in all U.S. counties (*156*). In general, CHSDA counties contain federally recognized tribal lands or are adjacent to tribal lands; however, rates restricted to these counties might not be generalizable to all AI/ANs because only 64% of AI/ANs live in a CHSDA county and because CHSDA counties tend to be in more rural areas and in Western states (*156*).

## Conclusion

Tobacco-associated cancer incidence can be reduced through efforts to prevent and control tobacco use and other comprehensive cancer control efforts focused on reducing cancer risk, detecting cancer early, and better assisting communities disproportionately impacted by cancer. Continued surveillance to measure characteristics within states can help identify populations with tobacco-associated disparities (*157*). Continued monitoring of cancer incidence rates at the local, state, and national levels can identify populations with high rates of tobacco-associated cancers and help evaluate the effectiveness of targeted tobacco control programs and policies.
